# METACOHORTS for the study of vascular disease and its contribution to cognitive decline and neurodegeneration: An initiative of the Joint Programme for Neurodegenerative Disease Research

**DOI:** 10.1016/j.jalz.2016.06.004

**Published:** 2016-12

**Authors:** METACOHORTS Consortium, Martin Dichgans, Joanna Wardlaw, Eric Smith, Vera Zietemann, Sudha Seshadri, Perminder Sachdev, Geert Jan Biessels, Franz Fazekas, Oscar Benavente, Leonardo Pantoni, Frank-Erik De Leeuw, Bo Norrving, Paul Matthews, Christopher Chen, Vincent Mok, Marco Düring, Will Whiteley, Kirsten Shuler, Alvaro Alonso, Sandra E. Black, Carol Brayne, Hugues Chabriat, Charlotte Cordonnier, Fergus Doubal, Emrah Duzel, Michael Ewers, Richard Frayne, Vladimir Hachinski, Mohammad Arfan Ikram, Frank Jessen, Eric Jouvent, Jennifer Linn, John O'Brien, Robert van Oostenbrugge, Rainer Malik, Bernard Mazoyer, Reinhold Schmidt, Luciano A. Sposato, Blossom Stephan, Richard H. Swartz, Meike Vernooij, Anand Viswanathan, David Werring, Koji Abe, Louise Allan, Francesco Arba, Hee-Joon Bae, Philip MW. Bath, Regis Bordet, Monique Breteler, Seong Choi, Ian Deary, Charles DeCarli, Klaus Ebmeier, Lei Feng, Steven M. Greenberg, Masafumi Ihara, Rajesh Kalaria, SanYun Kim, Jae-Sung Lim, Richard I. Lindley, Gillian Mead, Alison Murray, Terry Quinn, Craig Ritchie, Ralph Sacco, Rustam Al-Shahi Salman, Nikola Sprigg, Cathie Sudlow, Alan Thomas, Martin van Boxtel, Jeroen van der Grond, Aad van der Lugt, Yuan-Han Yang

**Keywords:** Dementia, Cerebrovascular disease, Small vessel disease, Neurodegeneration, Cohorts, Survey

## Abstract

Dementia is a global problem and major target for health care providers. Although up to 45% of cases are primarily or partly due to cerebrovascular disease, little is known of these mechanisms or treatments because most dementia research still focuses on pure Alzheimer's disease. An improved understanding of the vascular contributions to neurodegeneration and dementia, particularly by small vessel disease, is hampered by imprecise data, including the incidence and prevalence of symptomatic and clinically “silent” cerebrovascular disease, long-term outcomes (cognitive, stroke, or functional), and risk factors. New large collaborative studies with long follow-up are expensive and time consuming, yet substantial data to advance the field are available. In an initiative funded by the Joint Programme for Neurodegenerative Disease Research, 55 international experts surveyed and assessed available data, starting with European cohorts, to promote data sharing to advance understanding of how vascular disease affects brain structure and function, optimize methods for cerebrovascular disease in neurodegeneration research, and focus future research on gaps in knowledge. Here, we summarize the results and recommendations from this initiative. We identified data from over 90 studies, including over 660,000 participants, many being additional to neurodegeneration data initiatives. The enthusiastic response means that cohorts from North America, Australasia, and the Asia Pacific Region are included, creating a truly global, collaborative, data sharing platform, linked to major national dementia initiatives. Furthermore, the revised World Health Organization International Classification of Diseases version 11 should facilitate recognition of vascular-related brain damage by creating one category for all cerebrovascular disease presentations and thus accelerate identification of targets for dementia prevention.

## 1 Introduction

Worldwide, nearly 36 million people are estimated to be living with dementia. This is expected to triple by 2050. Cerebrovascular disease causes up to 45% of all dementias alone or in conjunction with Alzheimer's disease (AD) [1], [2]. Despite vascular risk factor reduction being an achievable target for public health intervention in many countries, and some recent evidence of success in preventing dementia [3], knowledge about vascular contributions to dementia remains modest.

Many studies, from the early 1990s onward [4], have demonstrated that cognitive impairment and dementia are both common and under-recognized after stroke [5]. The concept of “vascular cognitive impairment” was introduced in 1994 [6], covering a spectrum of cognitive impairment after stroke to cognitive impairment in association with otherwise asymptomatic cerebrovascular disease. The most common vascular contributor to dementia is cerebral small vessel disease (SVD) [7], a condition that affects perforating vessels, thence white and gray matter, and accelerates neurodegenerative processes. Vascular dementia reflects the global effects of vascular disease on the brain, not just of multiple individual infarcts. [8], [9] It results in stroke, cognitive decline and dementia, plus neuropsychiatric symptoms, gait, balance [8], [9], and continence problems [10], necessitating a larger framework for targeted, comprehensive studies [11].

**Fig. 1:**
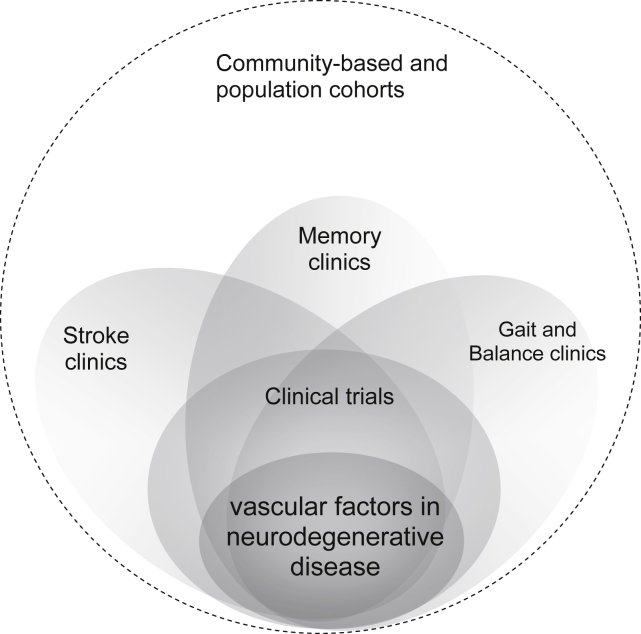
Approaches to tackling vascular factors in neurodegenerative disease. The challenge is to integrate the different clinical presentations when attempting to recognize more completely the interactions between vascular disease and neurodegeneration and thence improve prevention and treatment.

In 2006, the National Institute for Neurological Disorders and Stroke and the Canadian Stroke Network convened a multidisciplinary research group to recommend standards for the study of vascular cognitive impairment [11]. In 2013 the Alzheimer's Association convened an expert working group, which summarized the state of vascular cognitive impairment science and identified areas where new knowledge is needed [12]. However, despite strong and unanimous evidence for the major burden of vascular cognitive impairment on both patients and their caregivers [13], most dementia research largely overlooks vascular disease as a cause. In part, this reflects that clinicians and researchers working on dementia, stroke, physical, or psychiatric manifestations are still too often segregated. “Stroke” and “dementia” (both syndromes, not pathological diagnoses) present to different clinical specialists (Fig. 1); stroke specialists under-recognize the cognitive impact of stroke, whereas dementia specialists under-recognize vascular inputs to dementia pathophysiology. This separation also affects research and funding initiatives, for example, vascular disease was rarely mentioned in a report on 169 European studies considered relevant to neurodegenerative disease research [14]. Better diagnostic criteria for the different cognitive profiles of vascular and AD are also needed [15].

The recognition of an important role for cerebrovascular disease in dementia opens major therapeutic opportunities. Vascular risk factor reduction and stroke prevention may already be reducing dementia incidence [3], [16]. Increased government and public concern about dementia, as well as better grouping of codes for different cerebrovascular disease presentations in the revised International Classification of Disease (ICD) codes version 11 (ICD-11, release 2018, http://www.who.int/classifications/icd/revision/en/), will help advance understanding of cerebrovascular disease and its impact on neurodegeneration.

Here, we report on an initiative funded by the JPND to promote efficient use of available data in which we identified information, relevant to vascular disease, available in different types of studies that could provide large, statistically robust, generalizable data sets, and create platforms for future mechanistic, epidemiological research, or clinical trials [17]. We used the exemplar survey data to identify major gaps in knowledge, methodological issues and suggest priority actions to advance the field.

## 2 Method

We convened a group of experts in stroke and cerebrovascular disease, AD, epidemiology, psychology, neuroimaging, and clinical trials (Appendix). We designed a survey aiming to capture information about data available in cohorts relevant to vascular contributions to neurodegeneration. A “relevant cohort” could comprise patients with stroke/transient ischaemic attack (TIA), or suspected cognitive impairment or dementia, or healthy subjects, from a hospital or geographical population, and would have information on vascular disease and/or risk factors, and one or more of the following: cognitive data, long-term outcomes (including physical function and mood), neuroimaging including biomarkers of vascular disease and/or neurodegeneration, physiological measures (e.g., blood pressure, vascular stiffness), or biomarker samples with a relevant vascular link. The study could be completed or ongoing, cross-sectional or longitudinal, observational, or a clinical trial. The minimum sample for inclusion was 50 participants, with no age or geographical limits.

The survey (Supplementary Material), designed for online delivery, sought information on whether participants were healthy or recruited from a stroke or memory or other relevant clinic or participated in a clinical trial. Data were collected on demographics, medical history, risk factors, cognition, brain imaging, physiological measures, comorbidities at inclusion, duration and frequency of follow-up, follow-up assessments, outcome events, whether the study was completed or ongoing, availability of bio- or genetic samples for further analysis, interest of investigators in data sharing, and whether approvals for sharing were already in place.

The survey was piloted by three members of the expert group (JMW, MD, and ES), before being distributed widely. It ran from November 15, 2014 until August 31, 2015 with updates for ongoing studies to November 2015. We initially invited participation from investigators of studies in Europe through the JPND Vascular Disease group, relevant studies listed in the JPND report [14], networks, and studies known to them, for example, Dementia Platform UK, the German Center for Neurodegenerative Diseases (DZNE), and “Constances” in France; however, investigators based in North America, Australasia, and the Asia Pacific Region also expressed interest and were included. We recognized that the survey was unlikely to capture all studies but aimed to capture a broad sample, particularly from the vascular disease perspective (including clinical trials), as these are under-represented in other dementia initiatives [14], [18], [19], [20].

We performed descriptive statistics and meta-analyses (random effects methods) [21]. For detailed analyses of cognition in relation to stroke, we used studies that collected data on stroke and cognition from subjects without prestroke dementia in community-based studies or in subjects with stroke (hospital-based post-stroke cognitive impairment cohorts). Responses were received from 68 investigators (some provided data on several studies). Samples were updated to include ongoing recruitment to December 01, 2015. Five incomplete responses were removed from further analysis (for full details, see Supplementary Material).

Fifty-five experts (Appendix) discussed the data to identify knowledge gaps requiring new data, implications of ICD-11 disease codes (http://www.who.int/classifications/icd/revision/en/), agree early targets for shared data analysis, and plan future analyses of existing data and new research initiatives.

## 3 Results

**Fig. 2:**
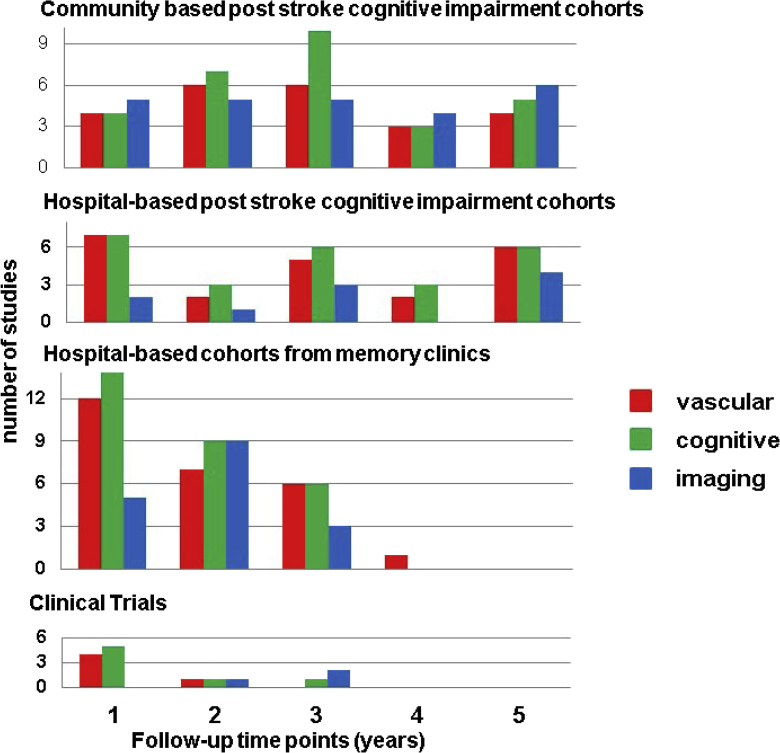
Duration of follow-up by study type and available information. Note some community-based studies have >5 years of follow-up.

The survey collected data on a total of 96 studies, including 167,064 participants, or 667,064 with UK Biobank [22] (186,000 and 686,000, respectively including target samples in ongoing studies; Table 1; Supplementary Table 1). The sample size ranged from 41 to 29,852 (excluding UK Biobank). The mean age was 72, range 15 to 106 years. There were 84 observational studies (11 cross sectional and 73 longitudinal) and 12 randomized clinical trials. The main types of studies overlapped (some recruited from several sources by various methods, Table 2) but, in general, most studies could be attributed to the following categories: community-based cohorts including population studies (32 studies, of which 28 were suitable for analyzing incident post-stroke dementia, sample size >600,000 subjects), hospital-based stroke clinics (i.e., stroke and TIA services, 26 studies, 12 suitable for analyzing incident post-stroke dementia, ∼4700 subjects), hospital-based memory clinics (15 studies, ∼20,000 subjects), and randomized clinical trials (12 trials, ∼20,000 subjects); 38 studies were ongoing (recruiting or long-term follow-up, Table 2). Some studies recruiting from mixed sources (Table 2, Supplementary Table 1) were not included in detailed analysis in the following section (or in Table 1), yet provide other relevant information (details in Supplementary Material). The longest duration of follow-up so far was more than 5 years (Fig. 2). Sixty-seven studies were based in Europe, 17 in North America, and 12 in the Asia Pacific Region (excluding 2 incomplete entries).

Most cohorts (∼86/96) did neither appear in the JPND report of 169 cohort studies [14] nor overlap by more than 20% with other recent initiatives, for example, Cohort Studies of Memory in an International Consortium (COSMIC) [18], Virtual International Stroke Trials Archive (VISTA) Cognition [22], or the Consortium of Studies of Post-Stroke Cognitive Decline and Dementia (STROKOG; [23]). This lack of overlap indicates a gap in information about vascular disease in neurodegeneration when viewed from “traditional” neurodegenerative perspectives and shows that there is a large amount of data available for sharing and meta-analyses if it could be brought together efficiently and effectively.

### 3.1 Data sources

#### 3.1.1 Community cohorts

Community-based (including population-based) cohorts are the best way to determine the relative contribution of vascular and other pathologies to the development of dementia and cognitive aging. They permit assessment of risk factor levels *before* the development of cognitive impairment or dementia, and hence, risk factor measurement is less likely to be affected by disease or its treatment. These cohorts often have repeated measures, starting in midlife, so, the impact of cumulative exposures, including during specific ages, can be explored. In contrast, clinical studies usually recruit from one extreme of the population distribution of vascular susceptibility; these studies may generate hypotheses that can be tested in population-based cohorts.

We gathered data from a representative sample of 32 community-based cohorts (Supplementary Table 1, Supplementary Fig. 1; 28 suitable for analysis) that defined the extent of vascular compromise, and structural brain injury in each person using serial brain imaging, and also measured global and domain-specific cognitive performance, mood, and physical function. Most studies prospectively followed their participants using health record linkage, questionnaires, or repeated examinations, to detect progression to cognitive impairment, stroke, or dementia. Most studies include biobanking, and a few have prospective postmortem brain banks. The community-based studies were either geographically population-based or recruited through advertisement and other strategies.

Most studies were conducted in the past three decades in North America and Europe, studied older participants (mean age 70 years), and used brain magnetic resonance imaging (MRI). Sample size ranged from around 100 to 500,000 participants (median: 1400; Q1–Q3: 400–9500), with imaging planned for ∼150,000. However, there are limitations. The imaging protocols, hence sensitivity to vascular pathology, varied, particularly in the older studies. Vascular risk factor assessment covered common risk factors, but interim TIA, clinical stroke, and stroke subtype were ascertained with varying degrees of rigor ranging from surveillance for incident events with direct participant examination and consensus review by study investigators, through medical records linkage, to self-reported events. Data on lipids, inflammatory markers, and renal function were missing from about half the studies. There was considerable variation in methods and timing used to assess vascular and cognitive outcomes. Data from a wider age span starting in youth to mid adulthood, diverse race, ethnic, and geographic origins, but using common protocols for cognition, physical function, minimum standard physiological measures, and MRI, are needed.

#### 3.1.2 Post-stroke cognitive impairment cohorts

In hospital-based series, about 20% of stroke patients have dementia after stroke, and the cumulative incidence of dementia after the first year is about 3% pa [24]. The survey identified 26 hospital-based cohorts recruiting from stroke/TIA ± other services (Table 2). Twelve had data suitable for analysis comprising studies that recruited patients presenting to hospital with ischemic stroke or TIA, followed longitudinally with cognitive and other measures. These studies collectively included >4134 subjects currently (planned >4700), and nearly 90% have structural neuroimaging. Most assessed educational attainment (although few assessed premorbid intelligence quotient), most used the Montreal Cognitive Assessment (MoCA) and all collected details of vascular disease and vascular risk factors (Supplementary Fig. 2). Of note, several cohorts include amyloid-positron emission tomography imaging, thus enabling assessment of the interactions between vascular and amyloid pathology in cognitive decline.

These data, if combined, would help overcome many gaps in knowledge remaining from previous post-stroke cognitive impairment studies [5], [25]: poor generalizability due to small sample and inclusion of high-functioning ischemic, nondysphasic, stroke patients, with an informant; or various entry restrictions, for example, inclusion of TIAs only, or first-only strokes, rather than any stroke. The variety of cognitive, physical, and physiological assessment tools and lack of prestroke cognition or contemporaneous mood data restrict comparisons. There is little information on concomitant AD pathology, or on biomarkers of vascular dementia, or pathology specimens to determine true proportions of vascular disease, and imaging acquisitions vary. As with population studies, agreed standards for future studies would increase research efficiency.

#### 3.1.3 Memory clinics

Patients attending a memory clinic represent a highly relevant population to study vascular contributions to cognitive decline and dementia. Most patients attending memory clinics have vascular lesions co-occurring with other pathologies, in particular, Alzheimer-type processes. However, there is limited evidence from longitudinal studies and randomized controlled trials (RCTs) in memory clinic-based cohorts to determine how much the vascular lesions contribute to cognitive profiles, predict prognosis, or should influence treatment. Extrapolation of observations from, for example, population-based studies, may not be valid because disease predictive factors in a relatively healthy population may not necessarily predict disease progression among individuals affected by the disease, or vice versa.

We identified 15 cohorts that included memory clinic patients (Table 2, Supplementary Fig. 3), some providing data only on memory [26], whereas others included other clinical presentations [27]. Cohort sizes were generally modest, with 8 of 15 cohorts including ≤200 patients, although baseline data are available on 19,144 patients of mean age of 73 years. All studies collected data on demographics, risk factors for vascular disease including education. Cognitive test results and neuroimaging (mostly MRI or computed tomography) were available from most patients in all cohorts. However, diagnostic criteria for mild cognitive impairment (MCI) and dementia, and its subtypes, varied across cohorts. Moreover, although most of the studies collected longitudinal data, both timing and content of follow-up varied substantially.

Unfortunately, substantial gaps in knowledge remain, such as the extent to which different pathologies may have differential prognostic impact in different stages of the dementia process. For example, larger studies at different disease stages across multiple clinics would help to determine if white matter hyperintensity (WMH) burden is indeed a stronger predictor of progressive brain atrophy in people with MCI and early AD than in later stages [28], [29]. Further studies are needed to determine how much co-occurring pathologies affect risk-benefit ratios of treatments that are typically used to reduce vascular risk, such as antithrombotic drugs. For example, randomized trials of aspirin in patients with AD observed rates of intracerebral hemorrhage that were much higher than those in people without AD [30].

#### 3.1.4 Physical function

Gait and balance disorders are common in elderly people, increasing rapidly from around 15% at the age of 60 years to >50% at age of ≥80 years [31], [32], [33]. They are often multifactorial and increase falls, institutionalization, and mortality [31], [32], [34]. For example, Parkinson's disease, impairs gait, and balance [35], but vascular cerebral disorders also disturb gait and may contribute to Parkinsonian symptoms [36], [37], particularly in SVD where gait is the second commonest symptom after cognitive disturbance [38], [39]. The Leukoaraiosis and Disability in the Elderly Study (LADIS) showed that gait and balance were correlated with WMH severity [8]. As gait represents a complex higher order form of motor functioning, impaired gait and cognition are closely intertwined [31].

However, gait and balance were rarely assessed in population-based or hospital-based post-stroke or memory studies. The survey found very limited information, but such details were poorly captured in the questionnaire. For example, none of five studies from general geriatric clinics mentioned recording gait, walking, or movement. Under “other,” no study mentioned these words either. Seven other studies mentioned “gait” (Supplementary Table 1, ABC1936, ABC1921, CASPER, ONDRI, PURE-MIND, RUN DMC, STRIDE) although we recognize that some studies that did not specifically mention gait do collect such data. This suggests that problems of gait and balance are under-recognized compared with other features of vascular neurodegeneration and hence are poorly assessed in vascular-focused clinics for older people, despite representing a major problem for older people, their families, hospitals, and social services.

#### 3.1.5 Clinical trials

Many acute stroke and stroke prevention RCTs have not collected cognitive data because of the following: (1) they focused on the physical consequences of stroke; (2) cognitive testing was considered too laborious and not applicable to participants whose vision, speech, or hand function were impaired; or (3) there was no informant, thus excluding significant proportions of patients from testing [40].

The survey collected data from 12 RCTs, testing treatments for acute ischemic or hemorrhagic stroke or secondary prevention of ischemic stroke (Supplementary Table 1). The sample size ranged from 41 to 4750, total current sample 20,035 (planned sample 22,314), of which 12,439 will have detailed neuroimaging. The mean participant age is 71.6 years, and 58.5% are men. Most trials consistently recorded baseline vascular risk factors including blood pressure, and outcomes such as recurrent vascular events and functional outcome assessed using the modified Rankin Scale. However, cognitive tests varied (eight used the mini-mental state examination [MMSE], one the MoCA, five the Telephone Interview of Cognitive Assessment (TICS), and two a more detailed assessment), none tested premorbid intelligence quotient although four trials recorded educational attainment, few assessed mood, and none corrected for imaging features such as WMH burden (a predictor of post-stroke cognitive impairment). The latest follow-up was at 12 months in all but two trials (latest assessment 24 and 36 months in one trial each), substantially shorter than that in the other study types (Fig. 2).

An individual patient data meta-analysis of cognition after stroke in these trials, which are typical of many stroke treatment or prevention trials, would be hampered by lack of consistency in cognitive measures and of long-term data, despite providing exemplary vascular risk factor and vascular outcome assessments. Inadequate attention has been given to assessing cognition after stroke in RCTs to date. Agreement on pragmatic and rapid ways to assess important cognitive domains such as executive function and processing speed, not just memory, and to correct for premorbid cognition, depression, and WMH burden on imaging, are essential to advance understanding of cognitive trajectories after stroke. There is an opportunity to progress by including cognitive tests in ongoing multicentre stroke trials where feasible as pragmatic methods are becoming available.

### 3.2 Methods for assessing vascular effects on cognition and neurodegeneration

Vascular disease requires different methods compared with other types of neurodegeneration or dementia research. Two key methods, which differ substantially in their requirements for vascular disease and neurodegenerative pathologies such as AD, are the assessment of neuroimaging and cognition. Integrated cerebrovascular disease codes are essential to bridge clinical presentations. All are discussed here.

#### 3.2.1 Imaging, protocols, and analysis methods

In the 1990s, landmark neuroepidemiological studies showed that clinically silent cerebrovascular lesions detected only on MRI, including lacunes and WMH of presumed vascular origin, were associated with cognitive impairment and an increased risk of future stroke and dementia [41], [42]. More recent studies have incorporated advanced imaging modalities that interrogate physiological and molecular changes—such as structural and functional connectivity with diffusion tensor imaging and functional MRI, cerebral perfusion, and molecular markers such as amyloid deposition—with larger sample sizes to increase statistical power for subgroup analyses and to predict clinical events.

Our synthesis of cohort studies identified many participants in community-dwelling settings and clinical studies on stroke who have cognitive data and undergone or will undergo neuroimaging, predominantly brain MRI (Table 1, Supplementary Table 1). We identified five major areas where there are currently limitations, gaps in knowledge, or unrealized opportunities for harmonization and collaboration of neuroimaging methods.

First, vascular lesion definitions and terminology require standardization, to enable cross-cohort comparisons and meta-analysis. This need has largely been met by the recent STandards for ReportIng Vascular ChangEs Neuroimaging (STRIVE) [44]; however, updates will become necessary for new neuroimaging methods that bring new imaging markers or increased sensitivity of known markers.

Second, to harmonize and compare findings across studies, full details of neuroimaging acquisition and analysis methods should be reported. New studies could adopt successful, validated methods recommended by STandards for ReportIng Vascular ChangEs Neuroimaging or used previously if the full methodological details were available. Imaging protocols could be shared through a single, publicly available website.

Third, reliability and accuracy of lesion classification would be improved through sharing of (exemplary) MRI data across cohorts with appropriate anonymization. For visual rating, a shared MRI repository could be used to train new raters using expert consensus as the gold standard. For computational analysis, for example, of MRI WMH, a repository could allow developers access to MRI images showing a range of lesions, from different vendors and field strengths, to derive or validate processing methods.

Fourth, there is great need for integrating information on vascular and neurodegenerative pathology from cohorts recruited through different settings, leveraging the expertise of stroke and dementia specialists in vascular and neurodegenerative disease. Integrating data across geographic and race/ethnic backgrounds would also help to more reliably identify and explore differences in subclinical vascular brain injury.

**Fig. 3:**
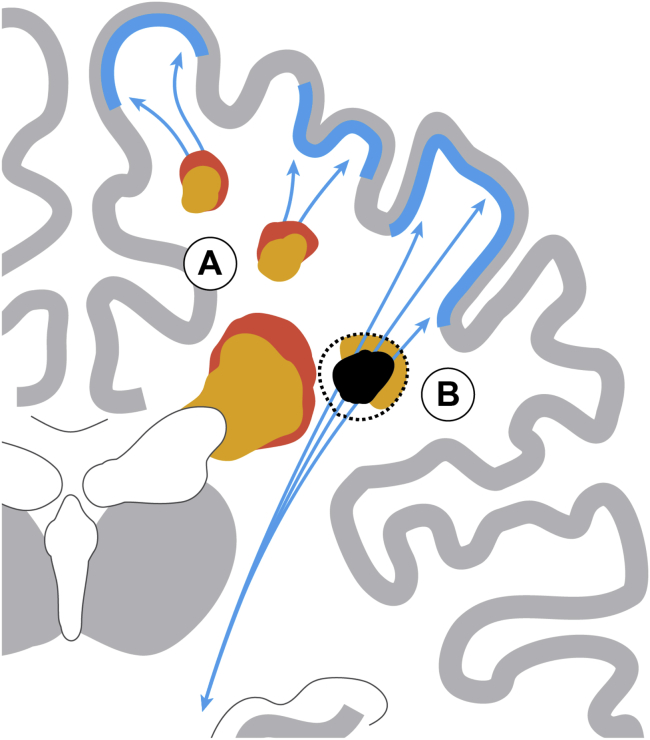
Dynamic effects of small vessel disease on the brain: (A) Periventricular and deep white matter hyperintensity (WMH; orange) can increase in size (red), occasionally shrink, and lead to atrophy of white matter. (B) Acute small subcortical infarcts (dashed black line) may cavitate and shrink (black area), develop into a WMH (orange) or disappear. Distant effects (blue) involve thinning of connected cortex and degeneration of projection fibers.

Fifth, more collaboration would enhance innovative methods for neuroimaging post-processing and data analysis. One example is the increasing emphasis on integrated data analysis to determine total SVD burden and effects on brain connectivity, neurodegeneration, and cognition (Fig. 3). Advances in machine learning and graph theory–based network analysis provide new opportunities to accelerate image analysis for large-scale studies. A multidisciplinary approach including neuroepidemiologists and clinical researchers on the one hand, and computer scientists, mathematicians and biomedical engineers on the other hand, is required.

#### 3.2.2 Cognition

There are particular challenges in the assessment of cognition in patients with cerebrovascular diseases, rendering detailed neuropsychological approaches (typically used in studies of dementia) impractical. Fatigue is common after stroke, limiting patient tolerance of prolonged tests. Patients may have dysphasia, impaired hand function, or visual deficits, making some tests impossible, even if comprehension is preserved. Depression and apathy are common in SVD and affect cognitive test performance. Many brief cognitive screening tests focus on memory, although vascular disease, in particular SVD, typically results in *subcortical* cognitive impairments, for example, loss of frontal and executive function [7], [43], and may occur in a stepwise manner reflecting sudden vascular events.

The survey shows huge variability in cognitive outcome measures in stroke/TIA patients (Table 1, Supplementary Material). About 25% of studies used a diagnosis of dementia or MCI, another 25% used a single cognitive test (most commonly MoCA, MMSE, or the revised Addenbrooke's cognitive examination); less than 10% assessed the premorbid (i.e., optimal early adulthood) cognitive status; and few assessed cognition immediately before the stroke. Of cognitive domains assessed in these studies, there was an almost equal distribution among memory, executive functions, reaction time, visuospatial function, with many accounting also for depression and anxiety.

Longitudinal observational studies included patients with different cognitive impairment or dementia diagnoses, but of note, about half the studies did not assign a specific dementia type and the diagnostic criteria for MCI, AD, vascular cognitive impairment (VCI), and dementia varied. The most commonly used criteria were the National Institutes of Aging-Alzheimer's Association for MCI and AD, *Diagnostic and Statistical Manual of Mental Disorders, IV Edition* for dementia, and the American Heart Association/American Stroke Association for VCI. However, with the exception of the widely used *Diagnostic and Statistical Manual of Mental Disorders, IV Edition* criteria for dementia, most studies used widely differing criteria for other categories of cognitive disorders, perhaps reflecting the difficulties and complexity of cognitive assessment in vascular disease. Most community-based and longitudinal observational studies used the MMSE, which is insensitive to vascular cognitive decline. A stepwise approach to determining the cognitive test approach to vascular disease is suggested in Table 3.

The importance of assessing cognitive profiles (without worsening the complexity of categorization) is emphasized by recent findings in SVD patients of memory loss (a cortical dysfunction) [7], [44], suggesting that SVD affects not just white and deep gray matter but also the (connected) cerebral cortex (Fig. 3) [45], [46], [44]. Lower connectivity within cerebral networks was associated with cognitive impairment and dementia [47], [48]; WMHs were associated with less brain activation in the frontal cortex on fludeoxyglucose-positron emission tomography [49], [50], [51]. Mood disorders and apathy are common after stroke, affect cognitive test performance (and more fundamentally, patient participation in research), and may reflect impaired connectivity. Integrating the results from different cohorts that investigate these underlying mechanisms and consequences is essential to understand the full impact of vascular disease on brain function and progression toward the complete spectrum of cognitive impairments.

#### 3.2.3 Relevance and mapping to next generation disease coding, ICD-11

Accurate and uniformly applied diagnoses are essential in health care and research. The World Health Organization has the main responsibility for the global classification systems, a core constitutional task. ICD-10 was published about 25 years ago. Major advances in the understanding of diseases have occurred since then, making ICD-10 outdated in several areas. The revision of ICD-10 into ICD-11 is currently in its final stage.

A major change from ICD-10 to ICD-11 is that cerebrovascular diseases will no longer be split across different chapters, but will constitute “one single section” in *Diseases of the Nervous System*. ICD-11 will also, for the first time, include definitions of all cerebrovascular diagnostic codes including definitions of transient ischemic attack and the different main types of stroke. It will also encompass cerebrovascular diseases not causing acute neurological dysfunction: *silent cerebral infarcts*, *cerebral microbleeds*, and *silent white matter abnormalities associated with vascular disease*. The term “silent” denotes that these entities have not caused acute neurologic symptoms (and hence are not “strokes”) but are important for brain function, affect prognosis, and should not be regarded only as incidental imaging findings.

The final ICD-11 classification is expected to be approved for governmental use by the World Health Assembly for release in 2018, but the prefinal beta draft is officially available at the World Health Organization website [52]. For several research purposes, it is recommended that the ICD-11 terminology and definitions be considered and may be applied already at this stage.

## 4 Discussion

Our initiative identified more than 90 cohort studies, including over 660,000 participants, many outside current neurodegeneration data initiatives, most with consent for data sharing, providing substantial scope for data mining. We acknowledge that our survey is incomplete. However, we consider it sufficiently representative to draw important conclusions. The segregation of stroke and dementia remains prevalent 10 years after the National Institute of Neurological Disorders and Stroke-Canadian Stroke Network (NINDS-CSN) standards [11], in spite of recognition that larger frameworks and better diagnostic criteria for dementias are urgently needed [1], [53]. Even among the survey respondents (likely “cerebrovascular disease enthusiasts”), there was a large gap between “stroke” and “dementia,” and sparse overlap with the JPND report [14]. Although stroke clinic–based studies and RCTs were trying to collect at least some cognitive data, the methods for testing cognition in such environments are suboptimal. Meanwhile, the memory clinic–based cohorts collected relatively little information on vascular disease. Undoubtedly, the role of vascular disease in neurodegeneration remains under-recognized, and under-funded [54], a situation that can no longer be justified: vascular risk factor reduction prevents stroke and may also prevent dementia [3] further evidenced by declining dementia incidence paralleling declining stroke incidence [16]. Fortunately, perspectives may be evolving. A recent call for new conceptual formulations of AD and dementia cites the need to account for mixed pathologies and known risk factors (many of which are also stroke risk factors) [55]. Furthermore, ICD-11 should help identify all cerebrovascular disease presentations. Standardized diagnostic workup and data collection would facilitate studies on diagnosis, prognosis, and treatment of vascular factors in a memory clinic setting, and similarly, studies of cognition, gait, and balance in a stroke clinic setting (Table 4). “Transnosological” research units, integrating specialists in neurovascular and neurodegenerative disorders would facilitate a global approach to dementia prevention.

These survey data provide a framework for addressing interactions between the two leading causes of cognitive decline and dementia: vascular disease and neurodegeneration. Epidemiological studies and RCTs are highly complementary when viewed as large data sets en masse. Observational studies indicate that the most critical period for elevated blood pressure with regard to cognitive decline is midlife, whereas blood pressure–lowering trials mostly included older patients with follow-up periods too short to detect an effect. Population studies inform hospital-based post-stroke dementia studies (and vice versa), often have repeated measures gathered over many years, enabling the impact of cumulative exposures, and of exposure during specific ages, to be explored. Important early life information is present in these studies, for example, birth weight, childhood cognition, socioeconomic data, for subjects now aged 50 to 70 years, enabling assessment of early life factors on cerebrovascular disease and dementia risk. Capturing information on both vascular and neurodegenerative disease from mixed sources improves generalizability (e.g., RUN DMC; FUTURE; Lund Stroke Registry, Supplementary Table 1). Combining studies that focus on populations in different epidemiological “windows” relative to the expression of disease enhances mechanisms' discovery and can relate systemic disease risk (obesity, metabolic, and cardiovascular disease) to cerebrovascular disease and dementia. Cohort meta-analyses would help clarify long-term event rates, their prediction, risk factors, and variation between populations and improve design of RCTs. Existing studies may provide well-phenotyped “trial ready” subjects with prospective consent for future research. The challenges involved in harmonization and analysis of large, diverse data sets are substantial (Table 4), but methods to overcome this are ongoing [56], and the potential rewards are huge. Work already ongoing as a result of this initiative is listed in Supplementary Table 2.

Agreeing on a unified cognitive assessment, which can be applied easily in cerebrovascular disease, is sensitive to relevant domains and relevant to patients, could have as much impact on identifying treatment to prevent VCI as the Rankin Scale [57] has had on finding acute stroke treatments: without the common language for functional outcome provided by the Rankin scale, it is arguable that stroke units, thrombolysis, hemicraniectomy, and thrombectomy would not have become guideline acute stroke interventions in as little as 20 years. Currently, most stroke RCTs, with few exceptions [58], do not assess cognition. The evaluation of cognition in stroke patients is complex, difficult, with no “best cognitive test”. The 2006 collaborative consensus [11] proposed three cognitive protocols with different lengths, one being a brief test for use in large observational studies and RCTs. Pragmatic, rapid, validated, tools sensitive to cognitive domains affected by vascular disease early on, are required, like the MoCA [59]. Online cognitive tests, for example, the UK Biobank Cognitive Testing Enhancement, are too complex for many stroke patients. In any case, a stepwise approach is needed to assess vascular effects on neurodegeneration (Table 3). Simple, sensitive, cognitive scales for use in telephone interviews, validated in patients with cerebrovascular disease, are needed (Table 4) [60].

Several large national initiatives will address dementia prevention in line with the 2013 G8 Dementia Summit. These draw largely on healthy or presymptomatic disease populations and offer new opportunities for systematic, prospective evaluations of people at scale, and often long-term sample biobanking, genetics, and imaging, enabling some powerful study designs. These include the Rhineland study (Germany, DZNE, n = 40,000), the Canadian Longitudinal Study on Aging (50,000 individuals), the Canadian Alliance for Healthy Hearts and Minds and Prospective Urban Rural Epidemiological MIND (PURE-MIND, 11,200 persons), and the UK Biobank [61] (500,000 people aged 40–70, 100,000 with detailed imaging). These long-term initiatives are complemented by several national and regional efforts to establish disease-based cohorts, for example, the Canadian Consortium on Neurodegeneration in Aging (1400 persons; AD, mixed dementia, MCI, and VCI), or to combine existing cohorts, for example, the DPUK [62], (29 UK community cohorts), Cohort Studies of Memory in an International Consortium (COSMIC) [18], Virtual International Stroke Trials Archive (VISTA) Cognition [22], and STROKOG [23]. Other regions should be encouraged and are creating large repositories of data—Asia Pacific Region, Central and America [63], Russia, Africa [64], and Australasia [18].

Governments, funders, and the public recognize the importance of sharing publicly funded data. Data from combined analyses of cohorts would provide larger samples, more robust data on individual cognitive and physical outcomes, and the interplay between brain and body to maintain healthy, active populations into old age. The 2015 World Stroke Proclamation on preventable dementias [1] has been endorsed by several international Alzheimer's, neurological, psychiatric, and heart associations. Funding for cerebrovascular disease research should more closely match that spent on dementia or cardiac disease [54]. Researchers should work together to operationalize assessment of vascular effects on neurodegeneration; stroke should move from “stroke-related” to “anything vascular related including cognition or other effects” [52]; and dementia should move from AD to “any disorder, arising in or outside the brain, that progressively diminishes cognitive function”.

## Figures and Tables

**Table 1:** Summary of types and amount of data available in cohorts recorded in the survey that were analyzed in detail

	Community-based cohorts	Hospital-based cohorts from stroke services	Hospital-based cohorts from memory clinics	Clinical trials	All
Number of studies					
Total/completed recruitment/completed follow-up	28/22/15	12/8/5	16/8/4	12/7/5	68/45/29
Current number of patients					
Total/with imaging∗	583.851/23.388	4.134/3.529	19.144/5.982	20.035/12.050	627.164/44.949
Planned sample size					
Total/with imaging data	>600.000/>150.000	4.702/4.289	21.353/8.153	22.314/12.439	∼655.000/∼172.000
Mean age (y)	71	70	73	71.6	72
Male sex (%)	46	56	51	58.5	46
Number of studies with					
Clinical diagnosis of stroke supported by neuroimaging					
MRI/CT/MRI or CT	6/1/4	5/4/3	4/0/2	3/2/6	18/7/15
Baseline information on risk factors					
Hypertension/diabetes mellitus/hypercholesterolemia/smoking/medication/education	26/26/22/26/27/27	12/12/12/12/12/11	16/16/16/16/15/16	10/10/10/9/8/4	64/64/60/63/62/58
Follow-up assessment					
After months 3/6/12/24/36/48/60	1/2/6/10/10/5/6	9/6/9/3/6/3/6	0/4/14/9/7/1/0	7/4/5/2/2/0/0	17/16/34/24/25/9/12
Functional outcomes					
mRS/BI/SIS/EuroQol/SF36/ADL or IADL	3/2/0/1/2/5	7/7/1/2/2/2	4/4/1/3/0/4	8/3/1/7/1/0	22/16/3/13/5/11
Vascular outcomes					
Stroke/TIA/MI/Vascular death	17/14/16/15	10/7/7/8	14/12/10/9	9/8/9/7	50/41/42/39
Cognitive outcomes					
MCI/Dementia/MoCA or ACE-R/MMSE/TICS/Memory/Executive/Reaction time/Visuospatial	7/15/4/17/16/18/15/16	7/9/7/10/8/7/4/7	8/11/6/12/14/14/10/14	1/2/1/8/6/4/4/4/4	23/37/18/47/44/43/33/41
Psychiatric outcomes					
Depression/anxiety	18/12	9/7	14/7	7/0	48/26
Mobility outcomes					
Gait/balance/manual dexterity	1/0/0	1/0/0	1/0/0	0/0/0	3/0/0
Criteria used for a diagnosis of MCI					
DSM-V/AHA or ASA/Petersen/NIA-AA/other	1/1/4/3/2	2/1/3/0/1	0/2/2/6/3	0/0/0/0/6	3/4/9/9/12
Criteria used for a diagnosis of dementia					
DSM-IV/DSM-V/ICD-10/other	7/3/3/3	5/1/1/0	10/0/0/3	1/0/0/6	23/4/4/12
Criteria used for diagnosis of vascular CI					
NINDS-AIREN/AHA or ASA/other	8/4/2	1/1/1	7/4/1	0/0/0	16/9/4
Criteria used for a diagnosis of AD					
NIA-AA clinical/DSM-V/DSM-V/NINCDS-ADRDA/other	5/5/0/2/6	1/4/1/0/0	8/2/1/4/2	0/0/0/0/0	14/11/2/6/8
Stored sample					
DNA/blood	26/20	7/6	13/14	3/3	49/43

Abbreviations: ACE-R, revised Addenbrooke's cognitive examination; AD, Alzheimer's disease; AHA, American Heart Association; AIREN, Association Internationale pour la Recherché et l'Enseignement en Neurosciences; ASA, American Stroke Association; CT, computed tomography; DSM, Diagnostic and Statistical Manual of Mental Disorders; ICD, International Classification of Disease; MoCA, Montreal Cognitive Assessment; MCI, mild cognitive impairment; MI, myocardial infarction; MMSE, mini-mental state examination; MRI, magnetic resonance imaging; NIA-AA, National Institutes of Aging–Alzheimer's Association; NINDS, National Institute for Neurological Disorders and Stroke; TIA, transient ischaemic attack; TICS, Telephone Interview of Cognitive Assessment.

NOTE. For studies not included in detailed analysis and full details of all studies, see Supplementary Table 1.

∗ Number includes UK Biobank of 500,000 recruited, 100,000 expected to have brain and other imaging. For 11 cross-sectional and 17 longitudinal studies not listed in Table 1, see Supplementary Material.

**Table 2:** Summary of 84 observational cohort studies by study setting

Setting	n
Observational studies	84
Cross sectional	11
Longitudinal	73
Community based	32
Via advertising	9
Population based	23
Hospital based for some recruits and community based for others	4
Stroke or TIA clinic	1
Stroke or TIA clinic and memory clinic	1
Memory clinic and general geriatric clinic	1
Other	1
Hospital/clinical based	37
Stroke or TIA clinic	19
Stroke or TIA clinic and memory clinic	2
Stroke or TIA clinic and memory clinic and general geriatric clinic	3
Stroke or TIA clinic and memory clinic and general geriatric clinic and any healthy volunteers	1
Stroke or TIA clinic and memory clinic and any healthy volunteers	1
Memory clinic	5
Memory clinic and general geriatric clinic	1
Other	5

Abbreviation: TIA, transient ischaemic attack

NOTE. Of 84 observational studies, 11 are cross sectional, and 73 are longitudinal. Data on 12 clinical trials are not included.

**Table 3:** Choosing tests to measure cognition in studies and trials dealing with vascular diseases: a step-based process

1) Decide to assess cognition (mandatory) 2) Decide whether to assess a) diagnostic outcome measure (e.g., dementia, cognitive decline, mild cognitive impairment) and/or b) cognitive measures (i.e., use domain-specific cognitive tests) 4) If b, decide which domains to assess, and 5) Which tests to use.

**Table 4:** Recommendations for research

Recommendation	Reason 1	Reason 2	Reason 3
General	Vascular and neurodegenerative pathologies are closely related; vascular pathology is an integral part of the pathological spectrum of AD, and vascular disease can play an important primary or secondary role to other pathology in neurodegeneration and dementia.	Secondary neurodegeneration due to vascular insults is an important contributor to accumulating structural brain damage and brain dysfunction.	Vascular damage can manifest as progressive cognitive, behavioral or sensorimotor dysfunction, that is, not just stroke.
Integrated approaches are needed	Vascular neurodegenerative disorders may present to many different clinics but are underpinned by a common vascular disorder.	These clinics should integrate to avoid overlooking the multifaceted effects of vascular disease on cognition, psychiatric symptoms, and physical function.	Clinical practice and research should assess risk factors, clinical, cognitive, imaging, and physical function.
Vascular disease is a dynamic and far-reaching process	Apparently small lesions that may precipitate clinical presentations have remote effects on other parts of the brain, which increase neurological and cognitive dysfunction.	Small lesions are also evidence of a global brain disease and should be treated as a progressive, global pathology.	Vascular lesions are not small, individually trivial lesions, without clinical meaningfulness.
Always assess vascular risk factors, disease burden, and outcomes	Cerebrovascular disease and AD share multiple risk factors, for example, smoking, hypertension, hyperglycemia, diabetes, and obesity. Vascular risk factors may have a greater impact in midlife than old age.	As an absolute minimum, routinely assess history of cerebrovascular, peripheral vascular, cardiovascular disease, blood pressure, smoking, exercise, occupation, and diet, blood lipids, blood glucose.	Pragmatic approaches can be suitable and avoid overburdening researchers and participants. Follow-up should continue long term.
Cognitive assessments should be performed in, and relevant to, vascular disease	Need to be applicable to vascular patients and the environment in which they typically present.	Adapted to reflect their specific cognitive deficits and physical limitations.	Assess executive function and processing speed in addition to memory.
	To avoid mistaking lifelong stable traits for late life change, routinely assess prior cognitive ability (or proxy measures, e.g., educational attainment) in any studies of cognition and vascular disease or other dementias.	Long-term outcome events, rates, and timings of decline in cognitive and physical function are needed to power clinical trials and inform patients and health services more effectively.	Socioeconomic factors have major influence on vascular disease beyond that attributed to vascular risk factors alone and should be assessed routinely.
Assess physical function across several domains routinely	Gait, balance, and continence are often affected and should be assessed routinely in suspected vascular cognitive impairment.	Simple tests such as “timed up and go” are valuable to assess physical function.	
Use standard, validated data collection that accounts for vascular disease	Agreed core standard data (clinical, cognitive, imaging, biomarkers, and so forth) and definitions would facilitate future data sharing and meta-analyses.	Agreed standards are available, for example, NINDS-CSN Vascular Cognitive Impairment Harmonization Standards; or STRIVE standards for neuroimaging.	Imaging can identify “silent” and symptomatic vascular disease if the right sequences are used.
Encourage postmortem brain tissue collection	Brain tissue from subjects well phenotyped in life, including brain regions commonly affected by vascular disease, are not widely available; storing samples frozen, and in paraffin, would facilitate protein, and gene as well as histological assessments.	More research is needed on the interaction of AD with cerebrovascular pathology, the consequences for function of brain networks, and ultimately how these pathologies evolve and combine to cause clinical consequences.	Tissue-imaging analysis of individual lesions is needed to understand pathological mechanisms.
Make better use of existing cohort data	Study registration and public availability of protocols would facilitate identification of novel and ongoing studies for meta-analyses.	Cohorts that assess cerebrovascular disease and include molecular imaging to assess AD pathology (e.g., by PET amyloid imaging) will help understand joint pathologies.	Open data initiatives and databanks would encourage sharing of existing cohort data.

Abbreviations: AD, Alzheimer's disease; PET, positron emission tomography.

## Appendix Authors

Study co-ordination, analysis and paper preparation: Martin Dichgans (1, Institute for Stroke and Dementia Research (ISD), Klinikum der Universität München, Ludwig-Maximilians-University LMU, Munich Germany; 2, German Centre for Neurodegenerative Diseases (DZNE, Munich), Munich Germany; and 3, Munich Cluster for Systems Neurology (SyNergy), Munich, Germany); Joanna Wardlaw (Centre for Clinical Brain Sciences, University of Edinburgh, UK); Eric Smith (University of Calgary, Canada); Vera Zietemann (Institute for Stroke and Dementia Research (ISD), Klinikum der Universität München, Ludwig-Maximilians-University, Munich, Germany); Sudha Seshadri (Boston University, USA); Perminder Sachdev (University of New South Wales, Australia); Geert Jan Biessels (UMC Utrecht, Netherlands); Franz Fazekas (Medical University of Graz, Austria); Oscar Benavente (University of British Columbia, Canada); Leonardo Pantoni (Azienda Ospedaliero Universitaria Careggi, Florence, Italy); Frank-Erik De Leeuw (Radboud University Medical Center Nijmegen, Netherlands); Bo Norrving (Lund University, Sweden); Paul Matthews (Imperial College, London, UK); Christopher Chen (National University of Singapore); Vincent Mok (The Chinese University of Hong Kong, China); Marco Düring (Institute for Stroke and Dementia Research (ISD), Klinikum der Universität München, Ludwig-Maximilians-University, Munich, Germany); Will Whiteley (University of Edinburgh, UK); Kirsten Shuler (University of Edinburgh, Edinburgh, UK).

Participants in working groups, survey, interpretation: Alvaro Alonso (University of Minnesota, USA); Sandra E Black (Sunnybrook Health Sciences Centre, University of Toronto, Canada); Carol Brayne (University of Cambridge, UK); Hugues Chabriat (Univ Paris Diderot, and DHU NeuroVasc, both at Sorbonne Paris Cité, UMR-S 1161 INSERM, F-75205 Paris; Hopital Lariboisiere, Dept of Neurology, F-75475 Paris, France); Charlotte Cordonnier (University Lille, Inserm, CHU, U1171 Degenerative and Cascular Cognitive Disorders, F-59000, Lille, France); Fergus Doubal (University of Edinburgh, Edinburgh, UK); Emrah Duzel (DZNE Magdeburg, Germany); Michael Ewers (Institute for Stroke and Dementia Research (ISD), Klinikum der Universität München, Ludwig-Maximilians-University, Munich, Germany); Richard Frayne (University of Calgary, Canada); Vladimir Hachinski (Rush University Medical Center, London, Ontario, Canada); Mohammad Arfan Ikram (Erasmus MC, Rotterdam, The Netherlands); Frank Jessen (University of Cologne, Germany); Eric Jouvent (Univ Paris Diderot, and DHU NeuroVasc, both at Sorbonne Paris Cité, UMR-S 1161 INSERM, F-75205 Paris; Hopital Lariboisiere, Dept of Neurology, F-75475 Paris, France); Jennifer Linn (University Hospital Dresden, Germany); John O'Brien (University of Cambridge, UK); Robert van Oostenbrugge (Maastricht UMC, Netherlands); Rainer Malik (Institute for Stroke and Dementia Research (ISD), Klinikum der Universität München, Ludwig-Maximilians-University, Munich, Germany); Bernard Mazoyer (University of Bordeaux, France); Reinhold Schmidt (Medical University Graz, Austria); Luciano A Sposato (Western University, London, Canada); Blossom Stephan (Newcastle University, UK); Richard H Swartz (Sunnybrook Health Sciences Centre, University of Toronto, Toronto, Canada); Meike Vernooij (Erasmus MC, Rotterdam, The Netherlands); Anand Viswanathan (Massachusetts General Hospital, Boston, USA); David Werring (UCL Institute of Neurology, London, UK).

Participants in the survey: Koji Abe (Okayama University Graduate School of Medicine, Dentistry and Pharmaceutical Sciences, Japan); Louise Allan ((Newcastle University, Newcastle, UK), Francesco Arba (University of Florence, Italy) on behalf of the VISTA Collaboration*; Hee-Joon Bae (Seoul National University Bundang Hospital, Republic of Korea (South Korea)); Philip MW Bath (University of Nottingham, UK); Regis Bordet (University Lille, Inserm, CHU, U1171 Degenerative and Vascular Cognitive Disorders, F-59000 Lille, France); Monique Breteler (German Center for Neurodegenerative Diseases (DZNE), Berlin, Germany); Seong Choi (Inha University Hospital, Incheon, Korea); Ian Deary (University of Edinburgh, UK); Charles DeCarli (University of California, USA); Klaus Ebmeier (University of Oxford, Warneford Hospital, Oxford, UK); Lei Feng (National University of Singapore); Steven M Greenberg (Massachusetts General Hospital, Boston, USA); Masafumi Ihara (National Cerebral and Cardiovascular Centre, Suita, Osaka, Japan); Rajesh Kalaria (Newcastle University, Newcastle, UK); SanYun Kim (Seoul National University Bundang Hospital, Republic of Korea (South Korea)); Jae-Sung Lim (Dept of Neurology, Hallym University Sacred Heart Hospital, Anyang, Republic of Korea (South Korea)); Richard I Lindley (University of Sydney, Australia); Gillian Mead (University of Edinburgh, UK); Alison Murray (University of Aberdeen, UK); Terry Quinn (University of Glasgow, UK); Craig Ritchie (University of Edinburgh, UK); Ralph Sacco (University of Miami, USA); Rustam Al-Shahi Salman (University of Edinburgh, UK); Nikola Sprigg (University of Nottingham, UK); Cathie Sudlow (University of Edinburgh, UK); Alan Thomas (Institute of Neuroscience and Newcastle University Institute for Ageing, Newcastle University, Newcastle, UK); Martin van Boxtel (Maastricht University, Netherlands); Jeroen van der Grond (Leiden University Medical Center, Netherlands); Aad van der Lugt (Erasmus MC, Rotterdam, The Netherlands); Yuan-Han Yang (Kaohsiung Medical University Hospital, Taiwan).

*VISTA Prevention Steering Committee: H-C Diener (Chair), S Davis, G Hankey, KR Lees, B Ovbiagele, C Weir.
