# Supplementary material for: METACOHORTS for the study of vascular disease and its contribution to cognitive decline and neurodegeneration: An initiative of the Joint Programme for Neurodegenerative Disease Research
Source: Alzheimers Dement. 2016 Dec;12(12):1235–49. doi: 10.1016/j.jalz.2016.06.004 (PMC5399602; doi:10.1016/j.jalz.2016.06.004)
Supplement: Supplementary Material [file mmc1.docx]

**Supplementary material**

**List of 11 cross-sectional and 17 longitudinal studies that were not included in main manuscript Table 1**

**Supplementary Table 1.** Studies participating in the survey

**Supplementary Table 2.** Ongoing research on shared data

**Detailed descriptions of survey findings by study type** including **Figures** (planned sample size, age and gender, outcomes collected, diagnostic criteria for different cognitive outcomes)

1 Population studies

2 Hospital-based cohorts recruited from stroke clinics

3 Hospital-based cohorts recruited from memory clinics

4 Clinical Trials

**References**

**Studies not included in main manuscript Table 1:** These studies had mixed data sources or other reasons for not fitting the study categories in Table 1. Their details are given in Supplementary Table 1 below.

Cross-sectional studies: n=11

| Lund Stroke Register | LSR |
| --- | --- |
| Leiden Longevity study | LLS |
| Quantitative Imaging Normative Cohort | |
| Epidemiology of Dementia in Singapore Study | EDIS |
| Multi-Centre Retina and Stroke Study | MCRS |
| Lacunar stroke study - Retrospective cohort | LAS retrospective |
| Cognition and depression in small vessel disease | none |
| Post-stroke triage "DOC": simple screening for Depression, Obstructive Sleep Apnea and Cognitive Impairment | DOC 1.0 |
| The Brain-Eye Amyloid Memory (BEAM) Study | BEAM |
| A Study of the clinical Utility, patient preference and cost benefit of SPECT and PET-CT brain imaging in the Evaluation and Diagnosis of Alzheimer’s Disease | SUSPECTED-AD |
| Feasibility and comparative accuracy of comnon cognitive screening tools in stroke | |

Longitudinal studies : n=17

| Mild Stroke Study 1 | MSS1 |
| --- | --- |
| RUNDMC | RUNDMC |
| The Follow -Up of Transient Ischemic Attack and Stroke Patients and Unelucidated Risk Factor Evaluation | FUTURE |
| Prognosis of InTraCerebral Haemorrhages | PITCH |
| Blood-brain barrier in cSVD cohort | Blood-brain barrier in cSVD cohort |
| Lacunar stroke project | Lacunar stroke project |
| High blood pressure and target-organ damage of the Brain | HYBRID |
| CADASIL | CADASIL |
| Maastricht Study cohort | Maastricht Study cohort |
| Edinburgh Stroke Study | ESS |
| DOC Utility | DOC Utility |
| CROMIS-2 (ICH) | CROPMIS-2 |
| CROMIS-2 (AF) | CROMIS-2 |
| Identifying Predictors of dementia with Lewy bodies in People with Mild Cognitive Impairment | LewyPro |
| Suita cohort | Suita cohort |
| Northern Manhattan Study | NOMAS |
| Cognitive Function and Ageing Study subsections | CFASdoppelt |

| **Name of cohort** | **Web link** | **Country** | **City** | **Aim** | | **Start date** | **Ongoing** | **Size** | **End date** | **Age at inclusion (mean; range)** | **Clinical measures** | **Biomarkers** | **Cognitive assessment** | | **MRI/neuroimaging** | **Genetic DNA possible** | | **Blood sample** | | **Consent for sharing** |  |
| --- | --- | --- | --- | --- | --- | --- | --- | --- | --- | --- | --- | --- | --- | --- | --- | --- | --- | --- | --- | --- | --- |
| **COMMUNITY VIA ADVERTISING OR OTHER METHOD OR COMMUNITY WITH POPULATION-BASED SAMPLING** | | | | | | | | | | | | | | | | | | | | |  |
| Aberdeen Birth Cohort of 1921 (ABC1921) | Not stated | UK | Aberdeen | Longitudinal study of cognitive ageing and health in a well-characterised population of old people | | 1997 | No | 275 current | 2007 | 77.5; 77-81 | Yes | Yes | Yes | | Yes | Yes | | Yes | | Yes |  |
| Aberdeen Birth Cohort of 1936 (ABC1936) | Not stated | UK | Aberdeen | Life course risk factors for late onset dementia | | 1999 | Unclear | 378 current | Ongoing dementia out-come ascertainment | 64.5; 64-66 | Yes | Yes | Yes | | Yes | Yes | | Yes | | Yes |  |
| Aberdeen Children of the Nineteen Fifties (ACONF) | http://www.abdn.ac.uk/childrenofthe1950s/ | UK | Aberdeen | To understand social and educational predictors of cognitive ability/disability | | 1962 | Yes | ~5000 current | 2019 | 61; 58-64 |  | Yes | Yes | | Yes in subgroup of 150+ ongoing | Yes | | Yes | | Yes |  |
| Ageing in a Community Environment Study (ACES) | Not stated | Singapore | Singapore | To describe the trajectories of cognitive and physical function decline among community-living Asian elderly. To identify the predictors of healthy and functional aging in a community environment. To formulate evidence-based prevention strategy for dementia and late life depression in Singapore. | | 2011 | Yes | 810 current; 1260 expected | 2016 | 68; 60-96 | No | Yes | Yes | |  | Yes | | Not stated | | Yes |  |
| Alberta Consortium on Promoting Healthy Brain Aging and Preventing Dementia (ABCs Study) | Not stated | Canada | Calgary | Serum and neuroimaging biomarkers that distinguish exceptional cognitive brain aging, cerebral amyloid angiopathy and Alzheimer's disease | | 2010 | Yes | 110 current; 325 expected | Not known | mean not provided; 60-90 | Yes | Yes | Yes | | Yes | Yes | | No | | Yes |  |
| ARIC-Neurocognitive Study (ARIC-NCS) | https://www2.cscc.unc.edu/aric/ | USA | Minnea-polis, MN; Jackson, MS; Forsyth County, NC; Washing-ton Co, MD | 1. Estimate prevalence of dementia and MCI by race and sex in individuals aged 70-89 2. Determine whether midlife (ages 45-64) vascular factors (risk factors and markers of macro and microvascular disease) predict dementia, MCI, and cognitive change. 3. Determine whether the associations between midlife vascular factors and dementia/MCI identified in Aim 2 differ by dementia/MCI subtype defined clinically or by MRI signs. | | 1987 | Yes | 10000 current | ongoing | 77; 68-90 (45-64 at first assess-ment in 1987) | Yes | Yes | Yes | | Yes | Yes | | Yes | | Yes |  |
| Austrian Stroke Prevention Family Study (ASPS-Fam) | Not stated | Austria | Graz | The Austrian Stroke Prevention Study is a single-center, prospective follow-up study on the cerebral effects of vascular risk factors in the normal elderly population of the city of Graz, Austria. The ASPS-Fam represents an extension of the Austrian stroke prevention study (ASPS). | | 2006 | Yes | 418 expected | 2015 | 65; 38-86 | Yes | Yes | Yes | | Yes | Yes | | Yes | | Yes |  |
| Austrian Stroke Prevention Study (ASPS) | Not stated | Austria | Graz | The Austrian Stroke Prevention Study is a single-center, prospective follow-up study on the cerebral effects of vascular risk factors in the normal elderly population of the city of Graz, Austria. | | 1991 | No | 2007 current | 2004 | 64; 45-90 | Yes | Yes | Yes | | Yes | Yes | | Yes | | Yes |  |
| Cambridge City over-75s Cohort (CC75C) | http://www.cc75c.group.cam.ac.uk | UK | Cam-bridge | Population-based study of dementia prevalence and incidence and changes in function (cognitive, physical and social) in older old age | | 1985 | No | 2610 current | 2013 | 81; 75-106 | Yes | Not stated | Yes | | No | Not stated | | Not stated | | Yes |  |
| Canadian Alliance for Healthy Hearts and Minds (Alliance) | http://fhs.mcmaster.ca/chanchlani/cvcd.html | Canada | Hamilton | 1.To understand the role of environment (such as community in which you live and work) and contextual factors (such as societal structure, activity, nutrition, smoking, and access to health services) on heart disease, stroke, memory and brain function. 2.To understand the culture and immigration related factors that affect health status in people belonging to ethnic groups such as South Asians, Chinese, African origin, and reserve-based Aboriginal people from across Canada. 3.To look for early signs of change in brain, blood vessels and the heart function with MRI. 4.To understand how changes in brain, blood vessels, and heart function relate to the social, environmental and contextual factors. | | 2014 | Yes | 2200 current; 9700 expected | 2020 | 55; 40-70 | Yes | Yes | Yes | | Yes | Yes | | Yes | | Yes |  |
| Canadian Longitudinal Study of Aging (CLSA) continues as Canadian Study of Health and Aging (CSHA) | [www.csha.ca](http://www.csha.ca) and www.clsa.ca | Canada | Hamilton, Montreal and Halifax | CSHA was planned in 1989 as a national longitudinal study to provide accurate statistics on the number of people who have dementia, including Alzheimer disease, in Canada. Note patients are recruited from clinics. | | 1991 | ongoing | >50,000 recruited | Not known (is a 3rd wave after 1997) | 45-76; 65-106 | Yes | Yes | Yes | | Yes | Yes | | No | | Yes |  |
| Cognitive Function and Ageing Study (CFAS) | www.cfas.ac.uk | UK | Cambridge | To estimate the prevalence and incidence of cognitive decline and dementia and the range of variation of those two measures throughout England and Wales. To determine the natural history of dementia, in particular the rate of progression of cognitive decline including the distribution of the interval between the identification of cognitive impairment and death. To evaluate the degree of disability associated with cognitive decline and the service needs this disability generates. | | 1991 | No | 13004 current | 1993; 2003 | mean not provided; 65-103 | Yes | Yes | Yes | | No | Yes | | Yes | | Yes |  |
| Cognitive Function and Ageing study II (CFAS II) | www.cfas.ac.uk | UK | Cambridge | This study builds on the design and infrastructure of initial CFAS study. The method of selection of the population base has been repeated. It's aim to observe the difference in the next generation of the older population, not only for the young-old (aged 65-84), but also for the oldest-old (aged 85 or over), who may be very different to the generation before them. | | 2008 | No | 7635 current | 2011 | mean not provided; 65-101 | No | Yes | Yes | | No | Yes | | No | | Yes |  |
| Community Diversity Cohort (UCDADC) | http://www.ucdmc.ucdavis.edu/alzheimers/ | USA | Sacramento | Assess risk factors for cognitive decline among a highly diverse population. | | 2000 | Unclear | 500 current; 500 expected | Not known(is a 3rd wave after 2010) | 74; 65-90 | Yes | Yes | Yes | | Yes | Yes | | Yes | | Yes |  |
| Epidemiology of Dementia in Singapore Study (EDIS) | http://www.nuhs.edu.sg/research/programmatic-research/major-research-programs/vascular-and-neurodegenerative-mechanisms-in-dementia.html | Singapore | Singapore | To examine the prevalence and risk factors of cognitive impairment and dementia in three major ethnicities of Singapore | | 2010 | Yes | 923 current; 1000 expected | 2015 | 71; 60-88 | Yes | Yes | Yes | | Yes | Yes | | Yes | | Yes |  |
| Framingham Heart Study (FHS) | https://www.framinghamheartstudy.org/ | USA | Boston, MA | To identify risk factors associated with cardiovascular disease including stroke; since inception, it has expanded to study dementia, Alzheimer Disease, osteoporosis, lung disease, sleep, cancer etc. Main study comprises Original cohort (Gen 1) examined since 1948 (32 times), the Offspring (Gen 2) their children and spouses examined since 1971 (9 times) and Gen 3 examined since 2001 (2 times). Also Omni 1 (1990 on) and Omni 2 (with Gen 3). Dates are for entire sample MRI sweeps. Some MRIs (e.g. stroke cases and controls) since 1990. | | 1999 | Yes | ~15,000 at start, ~8800 alive current | Not known (is a 3rd wave after 2050) | mean not provided; 20-105 | Yes | Yes | Yes | | Yes | Yes | | Yes | | Yes |  |
| Identifying Predictors of dementia with Lewy bodies in People with Mild Cognitive Impairment (LewyPro) | Not stated | UK | Newcastle upon Tyne | To identify biomarker (Imaging, blood and CSF samples) and clinical predictors of DLB in people with MCI and symptoms of Lewy body disease | | 2013 | No | 52 current; 110 expected | Not stated | 76; "60-0" | Yes | Yes | Yes | |  | Yes | | Yes | | Yes |  |
| Leiden Longevity study (LLS) | Not stated | The Netherlands | Leiden | Derive ageing markers | | 2009 | No | 600 current | 2011 | 66; 45-85 | Yes | Yes | Yes | | Yes | Yes | | Yes | | Yes |  |
| Lothian Birth Cohort 1936 (LBC1936) | www.lothianbirthcohort.ed.ac.uk | UK | Edinburgh | To find the determinants of non-pathological cognitive and brain ageing. | | 2004 | Unclear | 1091 current | Ongoing; 4^th^ wave in field until 2016 | 70, followed at 73, 76, 79 | Yes | Yes | Yes | | Yes | Yes | | Yes | | Yes |  |
| Lothian Birth Cohort 1921 (LBC1921) | www.lothianbirthcohort.ed.ac.uk | UK | Edinburgh | To study the determinants of non-pathological cognitive ageing. | | 1999 | Unclear | 550 current | Ongoing; 6^th^ wave in field til 2016 | 80 | Yes | Yes | Yes | | Yes | Yes | | Yes | | Not stated |  |
| Maastricht Ageing Study (MAAS) | http://maastrichtagingstudy.nl | The Nether-lands | Maastricht | Longitudinal study of cognitive ageing trajectories in adulthood; full assessments at 0, 6 and 12 years, questionnaires only at 3 and 9 years. | | 1993 | Main study ended; substudy ongoing with cognitive and MRI for BBB in 2016-17 | 1823 current | 2008 | 52; 24-81 | Yes | No | Yes | | Yes | Yes | | Yes for 838 subjects | | Yes |  |
| Newcastle 85+ Study | http://research.ncl.ac.uk/85plus/aboutourproject/ | UK | Newcastle | The Newcastle 85+ Study aimed to: â€¢ assess the spectrum of health in the oldest old â€¢ examine the associations of health trajectories and outcomes with biological, clinical and social factors as the cohort ages â€¢ identify factors which contribute to the maintenance of health and independence â€¢ advance understanding of the biological nature of human ageing | | 2006 | No | 851 current | 2012 | 85; 84-86 | Yes | Yes | Yes | | No | Yes | | Yes | | Yes |  |
| Northern Manhattan Study (NOMAS) | Northernmanhattanstudy.org | USA | New York City | Examine race/ethnic disparities in stroke risk. A sub study focused on MRI markers of subclinical cerebrovascular disease and their association with cognitive performance and decline. | | 1993 | Yes | 3298 current | 2019 (in a 3rd wave after 2015) | 68; 40-104 | Yes | Yes | Yes | | Yes | Yes | | Yes | | Yes |  |
| PREVENT Research Programme (PREVENT) | Not stated | UK | London | To identify risk factors for dementia and stratify risk to work towards disease modification. | | 2014 | Yes | 97 current; 250 expected | 2019 | mean not provided; 40-59 | Yes | Yes | Yes | | Yes | Yes | | Yes | | Yes |  |
| Prospective Urban Rural Epidemiological MIND substudy (PURE-MIND) | http://www.ucalgary.ca/esmithresearch/projects/pure-mind | Canada | Calgary | Risk factors and consequences of covert brain infarcts in middle aged participants | | 2010 | Yes | 1150 current; 1500 expected | 2018 | 58; 40-75 | Yes | Yes | Yes | | Yes | Yes | | Yes | | Yes |  |
| Quantitative Imaging Normative Cohort | Not stated | Canada | Calgary | Provide normative data for quantitative MR imaging techniques (T1 morphology, T1 and T2 relaxometry, QSM, ASL, perfusion and permeability with DCE*, rsfMRI*). *=planned in subsequent phases (2015). Study design is currently single centre at 3 T. Quantitative acquisition approaches are generalizable to many neuroimaging studies but were developed and are funded by a CIHR grant on small vessel disease. Multi-phased stud, which allows updates, additions and deletions to the protocol at each phase. Target group is normal individuals w/o neurological or cardiovascular disease between 20 and 89 years. MoCA >26 required for inclusion. | | 2013 | Yes | 230 current, 500 expected | ongoing | 45; 20-79 | No | No | Yes | | Yes | No | | No | | Yes |  |
| Rhineland Study | Not stated | Germany | Bonn | To investigate modifiable and non-modifiable causes of neurodegenerative and neuropsychiatric diseases - To find biomarkers/(multimodal) biomarker profiles To identify individuals at risk for neurodegenerative or neuropsychiatric disease, who might benefit from preventive interventions - To investigate (determinants of) normal and pathological brain structure and function over the adult life course | | 2015 | Yes | 25000 expected | Not stated | 60; 30-100 | Yes | Yes | Yes | | Yes | Yes | | Yes | | Yes |  |
| Suita cohort | http://www.epi-c.jp/archives/e016.html (only Japanese) | Japan | Suita, Osaka | The Suita Study is a population-based cohort study of CVD in Suita City, Osaka, Japan conducted by the National Cerebral and Cardiovascular Center (NCVC). Suita City is an urban area with its population being about 350,000. While 70+% of people in Japan live in an urban area, the Suita study is the only population-based study in urban area in Japan. | | 1989 | No | 6485 current | 1996 | mean not provided; 30-79 | Yes | Yes | No | | No | Yes | | Yes | | Yes |  |
| The Rotterdam Study (RS) | http://www.erasmus-epidemiology.nl/research/ergo.htm | The Netherlands | Rotterdam | The primary aim of the Rotterdam Study is to study causes and consequences of age-related diseases. | | 1990 | Yes | 14926 current 18926 expected | 2017 | 64; 45-103 | Yes | Yes | Yes | | Yes | Yes | | Yes | | Yes |  |
| Three City Study (3C) | www.three-city-study.com/ | France | Bordeaux | Estimate the contribution of vascular factors on the risk of dementia | | 1999 | No | 9294 current; 4000 expected | 2003 | 74; 65-101 | Yes | Yes | Yes | | Yes | Yes | | Yes | | Yes |  |
| UK Biobank (UKB) | www.ukbiobank.ac.uk | UK | Oxford; UK wide | To enable statistically well powered studies of the genetic, lifestyle and environmental determinants of diseases of middle and old age, including vascular disease, cancer, arthritis, dementia and other neurodegenerative conditions | | 2006 | Yes | 500000 current; 500,000 expected | longerm | 57; 40-69 | Yes | Yes | Yes | | Yes | Yes | | Yes | | Yes |  |
| Utrecht Diabetic Encephalopathy study 2 (UDES2) | Not stated | The Netherlands | Utrecht | Study the impact of diabetes on brain structure and cognition. Focus on microvascular lesions 7T MRI and connectivity DTI | | 2010 | Yes | 110 current | 2016 | 70; range not provided | Yes | No | No | | Yes | No | | Not stated | | Yes |  |
| Whitehall II Oxford MRI substudy (WIIOx) | <http://www.biomedcentral.com/1471-244X/14/159> | UK | Oxford (London) | The programme is an ongoing longitudinal study of factors influencing health and cognitive function. 25 year antecedent vascular and metabolic risk trajectories and morbidity, physical and mental activity, and 15-year memory decrement, depression, genotype, and measured resilience will be used to model brain changes in 800 subjects. The presence of detailed and frequently sampled cohort data in the Whitehall II study has a unique prospective analysis of effects of socio-demographic, physical and behavioural factors on brain integrity and expression of common health and cognitive outcoms | | 2012 | Yes | 650 current; 800 expected | 2016 | 70; 55-90 | Yes | Yes | Yes | | Yes | Yes | | Yes | | Yes |  |
| **HOSPITAL/CLINIC BASED** | | | | | | | | | | | | | | | | | | | | | |
| **Stroke or TIA clinic** | | | | | | | | | | | | | | | | | | | | | |
| Blood-brain barrier in cSVD cohort |  | The Nether-lands | Maastricht | To determine the role of BBB permeability in cSVD | 2012 | | Yes | 60 current; 90 expected | 2016 | 65; 45-80 | Yes | No | Yes | | Yes | No | | Not answered | | Yes | |
| CASPER study (CASPER) |  | The Netherlands | Maastricht | To study the cognitive and mood disturbances after stroke (early and late) | 2013 | | Yes | 75 current; 250 expected | 2017 | 67; 18-90 | Yes | Yes | Yes | | Yes | Yes | | Yes | | Yes | |
| Clinical Biological and Pharmacological Factors Influencing Stroke Outcome (BIOSTROKE) |  | France | Lille | The Biostroke cohort was initiated to identify the clinical, biological and pharma-cological factors influencing the short-term (3 months) severity of stroke. In a second step, we perform long-term (5 years) follow-up of patients to identify the factors predicting cognitive disorders. This should allow to: (i) identify the clinical, biological and pharmacological factors which influence post-stroke dementia; (ii) study the effect of specific drug classes (lipid-lowering drugs, anti-hypertensive drugs, antidiabetic drugs) on specific or non-specific biological markers; (iii) study the relationship between vascular event and pre-existing cognitive disorders. | 2005 | | No | 471 current | 2014 | 67; 53-80 | Yes | Yes | Yes | | Yes | Yes | | Yes | | Yes | |
| Cognition and depression in small vessel disease |  | Serbia | Belgrade | To analyze predictors of cognitive decline and late-onset depression in patients with cerebral small vessel disease | 2000 | | Yes | 454 current; 1000 expected | 2017 | mean not provided; 18-90 | Yes | Yes | Yes | | Yes | No | | No | | Yes | |
| Determinants/Mechanisms of Dementia After Stroke (DEDEMAS/ DEMDAS) |  | Germany | Munich | To identify and characterize determinants of cognitive impairment post stroke. | 2011 | | Yes | 192 current; 600 expected | 2021 | 71; 44-89 | Yes | Yes | Yes | | Yes | Yes | | Yes | | Yes | |
| DOC Utility |  | Canada | Toronto, Missassauga, London, Hamilton, Ottawa, Thunder Bay | To determine whether baseline screening for Depression, Obstructive Sleep Apnea and Cognitive Impairment can add to known clinical and demographic risk factors to predict the occurrence of a composite negative outcome (any of: recurrent stroke, myocardial infarction, death, or admission to a long-term care (LTC) / complex continuing care (CCC) facility) within one year of screening, in stroke prevention clinic patients. | 2014 | | Yes | 2000 current; 8000 expected | 2018 | 66; 15-100 | Yes | No | Yes | | No | No | | No | | New Applic-ation for anonymised will be required | |
| Lacunar stroke project |  | The Netherlands | Maastricht | To study differences in the two types of lacunar stroke | 2003 | | No | 170 current | 2012 | 64; 40-87 | Yes | Yes | Yes | | Yes | Yes | | Yes | | Yes | |
| Lothian INtraCerebral Haemorrhage Pathology Imaging and Neurological outcome study (LINCHPIN) | www.rush.ed.ac.uk | UK | Edinburgh | Investigate the underlying cause of ICH; sampled from a geographic population | 2010 | | Yes | 267 current; 350 expected | 2016 | 80; 45-101 | Yes | No | Yes | | Yes | Yes | | Not answered | | Yes | |
| Mild Stroke Study 1 (MSS1) |  | UK | Edinburgh | To assess differences in blood brain barrier permeability and retinal vasculature in patients with lacunar stroke and controls with mild cortical stroke. | 2005 | | No | 250 | 2011 | 68; 40-90 | Yes | Yes | No | | Yes | No | | No | | Yes | |
| Mild Stroke Study 2 (MSS 2) |  | UK | Edinburgh | To determine blood-brain barrier permeability in SVD; to determine clinical, functional and imaging outcomes at 1 year; cognition at 1 and 12 months; clinical, cognitive, physical and functional outcomes at 3 years after lacunar and mild cortical ischaemic stroke. | 2010 | | Unclear | 264 current | 2015 | 67; 36-92 | Yes | Yes | Yes | | Yes | Yes | | No | | Yes | |
| Post-stroke triage "DOC": simple screening for Depression, Obstructive Sleep Apnea and Cognitive Impairment (DOC 1.0) |  | Canada | Toronto | To determine whether simple, evidence-based, screening can be quickly and feasibly implemented (>85% of patients in an average of <6 minutes) in large-volume urgent TIA/stroke clinics to identify individuals at high-risk for the three most common and devastating post-stroke co-morbidities (depression, obstructive sleep apnea and cognitive impairment) as well as determine the diagnostic characteristics of the DOC screen compared to gold standard assessments for these conditions. | 2012 | | No | 1504 screened; 418 Cog current | 2014 | 66; 15-100 | Yes | No | Yes | | No | No | | No | | Yes | |
| SNUBH single center prospective VCI cohort |  | Republic of Korea (South Korea) | Seoul | 1. To identify cognitive trajectories of post-stroke cognitive impairment 2. To investigate the clinical and neuroimaging predictors for the conversion of post-stroke dementia 3. To investigate the feasibility of VCIHS-NP in a longitudinal study for post-stroke survivors cohort | 2007 | | Yes | 971 current; "undetermined" expected | 2050 | 69; 22-97 | Yes | Yes | Yes | | Yes | No | | Yes | | Yes | |
| STroke Registry Investigating cognitive DEcline study (STRIDE) |  | China | Hong Kong | To investigate the mechanisms of early and delayed cognitive decline after stroke/TIA. | 2009 | | Unclear | 1013 | 2015 | 70; 24-98 | Yes | Yes | Yes | | Yes | Yes | | Yes | | Yes | |
| Study of Factors Influencing Post-stroke Dementia (STROKDEM) |  | France | Lille | STROKDEM Study aims to identify prognostic factors (clinical and lesional, biological and genetic, pharmacological, hygienic-dietetic or social) that positively or negatively influence the onset of dementia following strokes, by prospective follow-up of patients without dementia who presented with a first stroke. The STROKDEM Study two secondary purposes: (I) the search for prognostic factors of cognitive decline without dementia; (ii) identify prognostic factors of various types of dementia (Alzheimer’s disease, vascular dementia, mixed dementia). | 2011 | | Yes | 180 current; 250 expected | 2020 | 65; 30-80 | Yes | Yes | Yes | | Yes | Yes | | Yes | | Yes | |
| The Follow -Up of Transient Ischemic Attack and Stroke Patients and Unelucidated Risk Factor Evaluation (FUTURE) |  | The Netherlands | Nijmegen | investigate causes and long term prognosis after stroke in young adults | 1980 | | No | ~ 500 current | 2014 | 44; 18-80 | Yes | Yes | Yes | | Yes | Yes | | Yes | | Yes | |
| **Memory clinic** |  |  |  |  |  | |  |  |  |  |  |  |  | |  |  | |  | |  | |
| Clinical Research Center for Dementia of South Korea (CREDOS) |  | South Korea | Incheon | To investigate clinical evidences of prevention, treatment, and care of Korean patients with dementia and apply those to treatment, education, and a policy decision. | 2005 | | Unclear | 15000 current | Not known (is a 3rd wave after 2015) | mean not provided; 40-90 | Yes | Yes | Yes | | Yes | No | | No | | Yes | |
| AMyloid imaging for Phenotyping LEwy body dementia (AMPLE) | not known, but details can be seen on UKCRN portfolio website (UKCRN I.D.14235) | UK | Newcastle-upon-Tyne | The aim of the current study is to undertake amyloid PET imaging in DLB and AD subjects. We wish to investigate the distribution of amyloid burden in DLB relative to AD and controls. We also want to determine the relationship between amyloid levels, clinical features of the disease, other imaging changes and subsequent clinical course. | 2013 | | Yes | 52 signed consent current; 80, DLB= 40, AD =-20, Con=20 expected | 2017 | 75; 63-89 | Yes | Yes | Yes | | Yes | Yes | | Yes | | Yes | |
| A Study of the clinical Utility, patient preference and cost benefit of SPECT and PET-CT brain imaging in the Evaluation and Diagnosis of Alzheimer's Disease (SUSPECTED-AD) |  | UK | Newcastle-upon-Tyne | The main aim was to investigate which clinically applicable functional imaging modality, positron emission tomography with fluorodeoxyglucose combined with CT (FDG-PET-CT) or single photon emission computed tomography using HMPAO (HMPAO or perfusion SPECT) has the greatest utility to a) distinguish those with neurodegenerative dementia at mild-moderate stages (Alzheimer’s disease (AD) and dementia with Lewy bodies (DLB)) from normal controls and b) distinguish between different subtypes of dementia (DLB and AD). | 2010 | | No | 109 signed consent current | 2011 | Mean and age range not provided | Yes | No | Yes | | Yes | No | | Not stated | | Yes | |
| DZNE longitudinal study on cognition and dementia (DELCODE) |  | Germany | Cologne | Prediction of Alzheimer's dementia in pre-MCI at-risk subjects AD-biomarker development | 2014 | | Yes | 130 current; 1000 expected | 2017 | 75; 60-85 | Yes | Yes | Yes | | Yes | Yes | | Yes | | Yes | |
| String Pearl of Pearls Initiative - Neurodegenerative disease (String of Pearls) |  | The Netherlands | Utrecht | Collect a national biobank with imaging data, blood and CSF from patients attending the memory clinics of each of the 8 university hospitals in NL | 2008 | | Yes | 800 current | Not known (began 2nd phase recruit-ment in 2012 and follow-up not done) | Mean and age range not provided | Yes | Yes | Yes | | Yes | Yes | | Yes | | Yes | |
| TRACE-VCI |  | The Nether-lands | Utrecht | Identify clinical subtypes and prognostic factors in patients with vci presenting at a memory clinic | 2010 | | Unclear | 941 current | 2015 | 67; 40-95 | Yes | Yes | Yes | | Yes | Yes | | Yes | | Yes | |
| Mixture of sources (Stroke/TIA clinics, memory clinics, geriatric clinics, acute stroke units, health volunteers, etc.) | | | | | | | | | | | | | | | | | | | | | |
| CAA without ICH cohort (CAA without ICH) |  | USA | Boston | To investigate the role of CAA pathology in individuals without intracerebral hemorrhage. | 2009 | | Yes | 70 current | 2020 | 75; 65-85 | Yes | Yes | Yes | | No | Yes | | Yes | | Yes | |
| CADASIL |  | France | Paris | Follow-up study of CADASIL patients | 2003 | | Yes | 250 current; 350 expected | None planned | 50; 18-90 | Yes | Yes | Yes | | Yes | Yes | | Yes | | No | |
| CROMIS-2 (AF) | www.ucl.ac.uk/cromis-2 | UK | London | Role of microbleeds and other SVD markers in predicting bleeding risk in patients treated with anticoagulants following cardioembolic ischaemic stroke. | 2011 | | Yes | 1500 | Recruitment end 2015; Final follow-up 2017 | 70; 40-95 | Yes | Yes | Yes | | Yes | Yes | | No | | Yes | |
| CROMIS-2 (ICH) | www.ucl.ac.uk/cromis-2 | UK | London | 1. To determine genetic variants associated with anticoagulant-associated and spontaneous ICH 2. To determine risk factors, associations and outcomes for anticoagulant-associated ICH compared to other spontaneous ICH. | 2011 | | Unclear | 1100 | 2015 | 75; 45-95 | Yes | Yes | Yes (CT in all; MRI in subset) | | Yes (in subset) | Yes | | No | | Yes | |
| Delirium and long-term cognitive impairment after stroke |  | UK | Edinburgh | To investigate the role of the HPA axis in delirium and long-term cognitive impairment after stroke | 2012 | | Unclear | 95 current | 2015 | Mean and age range not provided | Yes | No | Yes | | Yes | No | | Not stated | | Yes | |
| early stroke screening cohort |  | UK | Glasgow | To assess the cross sectional and prospective test accuracy of short mood / cognitive screening tools for detection of clinical mood disorder in an acute (<3/7) stroke population | 2013 | | No | 69 current | 2013 | 71; age range not provided | Yes | No | Yes | | No | No | | Not stated | | Yes | |
| Edinburgh Stroke Study (ESS) | not currently active | UK | Edinburgh | Study the epidemiological differences between and prognosis of different stroke subtypes | 2002 | | No | 2000 current | 2007 | 70; 18-100 | Yes | Yes | No | | Yes | Yes | | Yes | | Yes | |
| Feasibility and comparative accuracy of common cognitive screening tools in stroke |  | UK | Glasgow | To assess the feasibility, acceptability, opportunity cost of three cognitive screening tools in a stroke setting | 2014 | | Unclear | 52 current | 2015 | 75; 40-93 | Yes | No | Yes | | Yes | No | | Not stated | | Yes | |
| Harmonization study | http://www.nuhs.edu.sg/research/programmatic-research/major-research-programs/vascular-and-neurodegenerative-mechanisms-in-dementia.html | Singapore | Singapore | Longitudinal study of vascular cognitive impairment and dementia by a) Improving the Diagnosis of Vascular Cognitive Impairment by Prospective Longitudinal Clinical Studies b) Dementia Neuroimaging using Multi-Modal Magnetic Resonance and Retinal Imaging | 2010 | | Yes | 415 current; 600 expected | Not known (is a 3rd wave after 2016) | 72; 50-95 | Yes | Yes | Yes | | Yes | Yes | | Yes | | Yes | |
| Heart Brain Connection Study cohort (HBC study) |  | The Netherlands | Maastricht | to determine the relation between hemodynamic changes in the brain and cognition | 2014 | | Yes | 10 current; 633 expected | 2018 | Mean and age range not provided | Yes | Yes | Yes | | Yes | Yes | | Yes | | Yes | |
| High blood pressure and target-organ damage of the Brain (HYBRID) |  | The Netherlands | Maastricht | to study association between blood pressure characteristics and cSVD in essential hypertension | 2004 | | Yes | 218 current; 1200 expected | 2016 | 52; 40-66 | Yes | Yes | Yes | | Yes | Yes | | Yes | | Yes | |
| Lacunar stroke study - Retrospective cohort (LAS retrospective) |  | Austria | Graz | To provide additional morphological MRI characteristics of recent small subcortical infarcts (RSSI) like longitudinal extension, volumes and maximal axial diameters in relation to different locations and MRI sequences (DWI and FLAIR) - Correlation of RSSI morphology, size, shape, location and volume with vascular risk factors and diseases (potential stroke etiologies like cardioembolic or atheroembolic sources) | 2008 | | No | 344 current | 2013 | 71; 25-92 | Yes | No | No | | Yes | No | | Not stated | | Yes | |
| Leukoaraisois And DISability (LADIS) study | http://www.ladis.unifi.it/ | Italy | Florence | The main aim was to evaluate age-related cerebral white matter hyperintensities (WMH) as independent determinant of the transition from healthy status to disability in elderly individuals. To this end, patients with WMH of different severity and no or mild disability were followed-up for a period of 3 years to determine the proportion changing to a more severe disability status. | 2001 | | No | 639 current | 2006 | 74; 65-84 | Yes | No | Yes | | Yes | No | | Not stated | | Yes | |
| Lund Stroke Register (LSR) | http://www.skane.se/webbplatser/skanes-universitetssjukhus/organisation-a-o/neurologiska-kliniken/forskning-inom-neuroomradet/lund-stroke-register/ | Sweden | Lund | Study epidemiology risk factors, and outcome in stroke | 2001 | | Unclear | 4516 patients, 960 controls current; 400 expected | Not known (recruitment and follow-up listed as note completed) | 73; 18-1402 | Yes | No | No | | Yes | Yes | | Not stated | | Yes | |
| Maastricht Study cohort | http://www.demaastrichtstudie.nl | The Nether lands | Maastricht | The Maastricht Study is an extensive phenotyping study that focuses on the etiology of type 2 diabetes (T2DM), its classic complications, and its emerging comorbidities. | 2010 | | Yes | 3400 current; 10000 expected | 2017 | Mean and age range not provided | Yes | Yes | Yes | | Yes | Yes | | Yes | | Yes | |
| Medical Imaging Trials NEtwork of Canada - C6 Amyloid project (MITNEC-C6) | www.mitnec.org | Canada | Toronto | Patients who have high burden of Periventricular White Matter Hyperintensities (pvWMH), irrespective of presentation to a the memory clinic or stroke prevention clinic, will have a higher likelihood of amyloid positive scans and will show a greater increase in amyloid deposition over one year. | 2014 | | Yes | 5 current; 150 expected | 2016 | 82; 60 minimum age, maximum not provided | Yes | Yes | Yes | | Yes | Yes | | Yes | | Yes | |
| Multi-Centre Retina and Stroke Study (MCRS) |  | Australia | Sydney | Our aim was to establish whether there was an association between changes in the retinal microvasculature in those with acute lacunar stroke that might provide clues to the pathology of cerebral small vessel disease. | 2005 | | No | 1321 current | 2008 | 67; age range not provided | Yes | No | No | | Yes | No | | Basic hospital results record-ed | | Yes | |
| Neuroimaging of Inflammation in Memory and Other Disorders (NIMROD) |  | UK | Cambridge | Where and to what extent is neuroinflammation associated with each of Alzheimer's disease, dementia with Lewy bodies, frontotemporal dementia, progressive supranuclear palsy, vascular dementia, late life depression and mild cognitive impairment? | 2013 | | Yes | 80 current; 120 expected | 2017 | mean not provided; 50-85 | Yes | Yes | Yes | | Yes | Yes | | Yes | | Yes | |
| Newcastle Cognitive Function after Stroke study (COGFAST) |  | UK | Newcastle upon Tyne | To examine incidence of cognitive impairment after stroke in an older cohort (>75 years) To examine predictors of cognitive decline To examine neuropathological correlates of cognitive decline in stroke survivors | 1999 | | Yes | 355 current | 2026 | 80; 75-94 | Yes | Yes | Yes | | Yes | Yes | | Yes | | Yes | |
| Ontario Neurodegenerative Disease Research Initiative (ONDRI) | www.ondri.ca | Canada | Toronto | The primary hypothesis is degenerative cognitive impairment can be caused by a heterogeneous group of disorders that can be explained, characterized and predicted using an integrated multimodality approach. The primary objective of the study is to use an Ontario-wide research consortium that will integrate a wide range of experimental, clinical, imaging and epidemiological expertise to specifically address the occurrence of degenerative cognitive impairment in the aging population. | 2014 | | Yes | 52 current; 150 expected plus other cohorts | 2018 | 67; 55-85 | Yes | Yes | Yes | | Yes | Yes | | Yes | | Yes | |
| Prognosis of InTraCerebral Haemorrhages (PITCH) |  | France | Lille | long term prognosis of ICH patients including cognitive decline/dementia | 2004 | | Yes | 560 current | 2019 | 72; 20-90 | Yes | No | Yes | | Yes | No | | Not stated | | Yes | |
| RUNDMC |  | The Nether-lands | Nijmegen | Causes and consequences of cerebral small vessel disease | 2006 | | Unclear | 503 current | Not known (is a 3rd wave after 2013) | 65; 50-85 | Yes | Yes | Yes | | Yes | Yes | | Yes | | Yes | |
| Superficial Siderosis in Patients with suspected Cerebral Amyloid AngiopathySuS (SuSPect-CAA) |  | Germany | Dresden | To prospectively evaluate if superficial siderosis is a predictor for future stroke and mortality in affected patients | 2011 | | Yes | 80 current; 200 expected | 2018 | 75; 55-95 | Yes | Yes | Yes | | Yes | Yes | | Yes | | Yes | |
| Sydney Stroke Study |  | Australia | Sydney | 1. Characterise the neuropsychological profile of stroke patients and track the course of their cognitive impairments over time. 2. Investigate determinants of, or associations of various factors with, post-stroke cognitive impairment, including dementia. | 1997 | | No | 252 current | 2005 | 72; 49-87 | Yes | Yes | Yes | | Yes |  | | Not stated | | Yes | |
| The Brain-Eye Amyloid Memory (BEAM) Study |  | Canada | Toronto | 1) To investigate three novel, non-invasive ocular measurements in a cross-sectional study of subjects with various neuro-degenerative dementias and subcortical ischemic vascular disease against standard cognitive assessments and brain imaging measures; and 2) To assess the potential utility of ocular assessments for early detection in the pre-dementia, i.e. the so-called Mild Cognitive Impairment (MCI) stage. | 2015 | | Yes | 320 expected | 2018 | 70; 50-90 | Yes | Yes | Yes | | Yes | Yes | | Yes | | Yes | |
| Vascular Cognitive Impairment Cohort (VCI) |  | USA | Boston | To understand the relationship between vascular risk factors and cognitive impairment in patients with subjective cognitive complaints and MCI. | 2009 | | No | 70 current | 2014 | 75; 65-85 | No | Yes | Yes | | Yes | Yes | | Yes | | Yes | |
| Vascular MCI Tuscany Study (VMCI-Tuscany Study) | http://www.vmci-tuscany.it/ | Italy | Florence | To estimate the net and multivariable effect in predicting the transition from VMCI to dementia studying a large set of both conventional and nonconventional clinical, neuroimaging, and biological markers of SVD | 2011 | | Unclear | 200 current | 2015 | 74; 54-89 | Yes | Yes | Yes | | Yes | No | | Yes | | Yes | |
| White matter hyperintensities in Alzheimer’s Disease (WMH IN AD) |  | Taiwan | Kaohsiung | Evaluating the effects of white matter lesion to the clinical course of Alzheimer's disease. A longitudinal survey to recruit all participants diagnosed as Alzheimer's disease | 2011 | | Unclear | 600 current; 700 expected | Not known (is a 3rd wave after 2013) | 78; 62-91 | Yes | Yes | Yes | | Yes | Yes | | Yes | | Yes | |
| **Clinical trials** | | | | | | | | | | | | | | | | | | | | |  |
| Efficacy of nitric oxide in stroke (ENOS) | http://www.enos.ac.uk/ | UK | Notting-ham | To determine if blood pressure lowering with a glyceryl trinitrate patch started within 48 hours of stroke improved outcome after acute stroke; to determine if patients should continue or stop antihypertensive drugs taken prior to the stroke. | | 2001 | No | 4011 final | 2014 | 70; (23-100) | Yes | Yes | Yes | Yes | | Yes | Yes | | Yes | |  |
| Exercise and vascular plasticity in metabolic syndrome (EnergI) | Not stated | Germany | Magde-burg | To assess the interaction of metabolic syndrome and amyloid deposition in old age in terms of their influence on vascular plasticity after exercise | | 2015 | Yes | 320 expected | 2019 | 70; 65-75 | Yes | Not stated | Yes | Yes | | Not stated | Not stated | | Yes | |  |
| FOCUS trial (FOCUS) | www.focustrial.org.uk | UK | Edinburgh | to determine if 20mg fluoxetine daily for 6 months reduces dependency at 6 months | | 2012 | Yes | 1110 current; 3000 expected | 2018 | 72; 18-90 | Yes | No | Yes | No | | No | No | | Yes | |  |
| Physical exercise and vascular plasticity (PEXVP) | Not stated | Germany | Magde-burg | Assess the effects of aerobic exercise on hippocampal perfusion in old age and individuals at risk of AD | | 2012 | Yes | 40 current; 150 expected | Not stated | 68; 62-78 | Yes | No | Yes | Yes | | No | No | | Yes | |  |
| PODCAST | http://www.podcast-trial.org | UK | Notting-ham | Randomised multi-centre trial of intensive versus guideline blood pressure lowering, and intensive versus guideline lipid lowering | | 2010 | No | 83 | 2014 | 74; 60-89 | Yes | Yes | Yes | Yes | | Yes | Yes | | Yes | |  |
| PROSPER | Not stated | The Nether-lands | Leiden | randomised trial pravastatin use | | 1999 | No | 4750 current | 2004 | 74; 70-83 | Yes | Yes | Yes | Yes | | Yes | Yes | | Yes | |  |
| Rapid Intervention with Glyceryl trinitrate in Hypertensive Stroke trial (RIGHT) | http://www.right-trial.org | UK | Notting-ham | Randomised single centre trial of glyceryl trinitrate versus sham in patients with ultra-acute stroke | | 2010 | No | 41 | 2012 | 70; 40-95 | Yes | Yes | Yes | Yes | | Yes | Yes | | Yes | |  |
| Rapid Intervention with Glyceryl trinitrate in Hypertensive Stroke trial-2 (RIGHT-2) | http://right-2.ac.uk/ | UK | Notting-ham | Randomised multicentre trial of glyceryl trinitrate versus sham in patients with ultra-acute stroke | | 2015 | Yes | 8 current; 850 expected | 2018 | 79; 55-91 | Yes | Yes | Yes | Yes | | Yes | Yes | | Yes | |  |
| Secondary Prevention of Small Subcortical Strokes (SPS3) | Not stated | Canada | Vancouver | Randomized multicenter trial for secondary stroke prevention in patients with lacunar strokes | | 2003 | No | 3020 current | 2012 | 63; range not provided | Yes | Yes | Yes | Yes | | Yes | Yes | | Yes | |  |
| TICH-2 | tich-2.org | UK | Nottingham | To assess whether tranexamic acid is safe and reduces death and dependency after hyperacute (within 8 hours of onset) spontaneous intracerebral haemorrhage. | | 2013 | Yes | 940 current; 2000 expected | 2017 | 69; 32-97 | Yes | No | Yes | Yes | | No | No | | Yes | |  |
| Triple Antiplatelets for Reducing Dependency After Ischaemic Stroke (TARDIS) | http://www.tardistrial.org/ | UK | Nottingham | To compare intensive versus guideline antipletelt therapy in patients with acute ischaemic stroke or TIA | | 2009 | Yes | 2943 current; 4100 expected | 2017 | 69; 50-97 | Yes | Yes | Yes | | Yes | Yes | | Yes | | Yes |  |
| Vitamins to prevent stroke trial (VITATOPS)^1^ | Not stated  (imaging substudy in VISTA-Cog) | Australia/ UK | Perth/ Glasgow | Determine whether the addition of B vitamin supplements to best medical and surgical treatments reduces the combined incidence of stroke, myocardial infarction and vascular deaths in patients with recent stroke or transient ischemic attack. | | 1998 | No | 430 current (imaging sub-study) – note 3089 pts have cognition | 2008 | 64; 25-100 | Yes | No | Yes | Yes | | No | No | | Yes | |  |

^1^VITATOPS trial total was 8164, of whom 3139 had MMSE during follow-up to a median of 2.8 years;[1] 430 participated in an MR imaging follow-up substudy.[2]

Studies identified after closure of the Survey include: the Brain and Gait Study (London Canada),[3,4,5,6,7] a population-based stroke and TIA incidence study in Central and South America,[8] and a study of cognitive impairment in stroke survivors in Nigeria.[9,10]

**Supplementary Table 2:** Ongoing research on shared data

|  | **Primary outcome** | **Covariate effects** |
| --- | --- | --- |
| **Cognition** | Dementia prediction scores | age, disease presentation, etc |
|  | Rate of cognitive decline stratified by dementia type diagnosis, | Pre-morbid IQ, vascular risk factors, age, presenting feature, etc |
|  | Influence of incident lacunes location | Pre-morbid IQ, vascular risk factors, age, presenting feature, etc |
|  |  |  |
| **Physical outcomes** | Recurrent stroke, dependency, death, cognitive impairment rates | vascular disease presentation type, age, vascular risk factors, vascular disease burden on imaging, etc |
|  | Incidence and prevalence of gait and balance problems | presenting feature, age, vascular risk factors, vascular disease burden on imaging, etc |
|  |  |  |
| **Imaging** | Evidence-based standards for image acquisition and analysis of SVD features | Advisory minimum standard, for quick to detailed imaging |
|  | Prevalence of SVD features in different geographical populations | age, vascular risk factors, vascular disease burden on imaging, etc |
|  | Incidence of new lesions and rates of progression of existing lesions | presenting feature, pre-morbid IQ, age, vascular risk factors, vascular disease burden on imaging, etc |
|  | Validity of the total burden of vascular disease scores | presenting feature, pre-morbid IQ, age, vascular risk factors, etc |
|  | Dynamic effect of subcortical lesions on cortical damage | Impact on cognition and physical decline |
|  | Haemorrhagic lesion burden | prevalence; distribution and risk factor influence |
|  | Do perivascular spaces predict WMH development? |  |
| **Lifestyle influences** | Effect of exercise on hippocampal volume |  |

**Population studies (Suppl Figure 1)**

The 2011 AHA/ASA scientific statement on vascular contributions to cognitive impairment and dementia defines vascular cognitive impairment (VCI) as “a syn­drome with evidence of clinical stroke or subclinical vascular brain injury and cognitive impairment affecting at least one cognitive domain”.[11] The entire spectrum of cognitive disorders is included, with all forms of cerebral vascular brain injury, ranging from mild injury perceived only with sensitive imaging techniques such as diffusion tensor imaging to conventional measures such as white matter lesions and lacunar infarcts and the entire spectrum of cognitive and mood changes from mild cognitive impairment to clinical dementia and including psychomotor slowing and depression. Impor­tantly, memory impairment is not a requirement for diagnosis because unlike Alzheimer disease, which in most cases begins in the mesial temporal lobe structures and affects hippocampal – and thus memory – function, vascular damage is topographi­cally heterogeneous and can involve deficits isolated to other domains.

Population-based cohorts have two major advantages: they show the entire range of vascular brain injury, without the referral bias inherent in hospital or clinic based cases and they permit assessment of risk factor levels before a clinical event. This is important for understanding routes to primary prevention and for understanding biology since physiological changes and initiation of treatment after an event can alter risk factor levels. Further population studies often have repeated measures, gathered over many years, starting in midlife so the impact of cumulated exposures and of exposure during specific ages (such as in midlife) can be explored. Hospital based studies represent one extreme of the population distribution. They can generate hypotheses based on the large number of disease events and these can be extended to population based cohorts. This approach is feasible because a number of studies of cardiovascular disease or normal cognitive aging have incorporated serial brain imaging and cognitive testing in the past 3 decades, and a few have additionally initiated brain banks to permit clinical-imaging–pathologic correlations. The initial studies were located largely in North America and Europe and include the Cardiovascular Health Study (a random sample of persons over age 65 at enrollment), the Atherosclerosis Risk in Communities Study (random sample of persons aged 45-65 at enrollment), the Framingham Heart Study (grandparental, parental and third generation participants drawn from a sample of persons living in Framingham, MA in 1948), the Age-Gene-Environment Study in Reykjavik, Iceland, the Rotterdam Study (recruited 3 waves of Rotterdam residents, aged >55, >55 and most recently >45 years), Three Cities (3C) Study in France, multiple UK based studies (Lothian Birth Cohorts, Newcastle 85+) and the Canadian Study of Health and Aging Suppl Table 1).

Altogether there are 28 suitable community based cohort studies, 8 of them community based via advertising or other methods (ACES, Alliance, UDES2, 3C, PREVENT, LBC1936, LBC1921, ABCs Study) and 20 with population-based sampling (FHS, Rhineland Study, CSHA, PURE-MIND, ACONF, Newcastle 85+ Study, UKB, ABC1936, ABC1921, LINCHPIN, CFAS, CFASII, WIIOx, MAAS, ASPS-Fam, ASPS, RS, UCDADC, ARIC-NCC, CC75C). Nineteen of these studies have more than one wave, 22 have already completed the recruitment of the first or only wave and 15 of them have also completed follow-up. Eight cohorts recruited any healthy volunteers, 17 cohorts selected participants from a geographical population, one cohort recruited from a stroke or TIA clinic, one is an occupational cohort and one is a primary care register. Eleven studies recruit patients with stroke or TIA, 4 studies specified no selection based on health status and all studies investigate stroke as an event during follow-up. Sample size is very diverse, ranging from around 100 to 500.000 patients (median: 1600; Q1-Q3: 359-9700), resulting in a planned sample size of over 600.000 patients. Imaging is planned for more than 150,000 patients. Mean age is 71 years and 46% of the patients are men (figure 1). Interim TIA, clinical stroke and stroke subtype were ascertained with varying degrees of rigor ranging from surveillance for incident events with direct examination of affected participant and consensus review by study investigators, through medical records linkage and review, to self-reported events. Baseline assessment covered relevant risk factors in almost all studies (e.g. hypertension, diabetes mellitus, smoking, medication, education). Hypercholesterolemia is missing in 6 studies and blood inflammatory markers and renal function in 15 studies. Seventeen studies performed MRI and one study a mix of CT and MRI at baseline. T1 is available in 17 studies, FLAIR in 15 studies, T2 in 14 studies, T2* in 8 studies and DWI in 11 studies. DNA is stored in 26 and blood in 20 studies. There is a large overlap in the methods used to measure vascular and cognitive outcome during follow-up (Supplementary Figure 1c). Five studies used linkage to other data sources to get data on the outcome. The others used specific follow-up time points measuring these outcomes and they are well matched (Figure 2 in main paper).

**Supplementary Figure 1 Population studies**


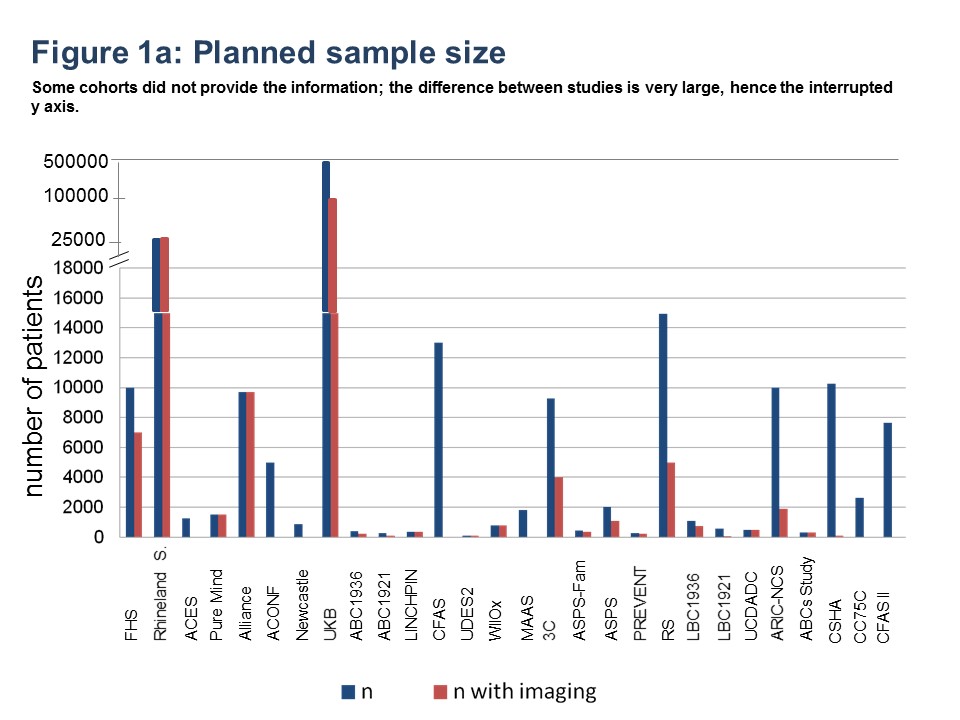

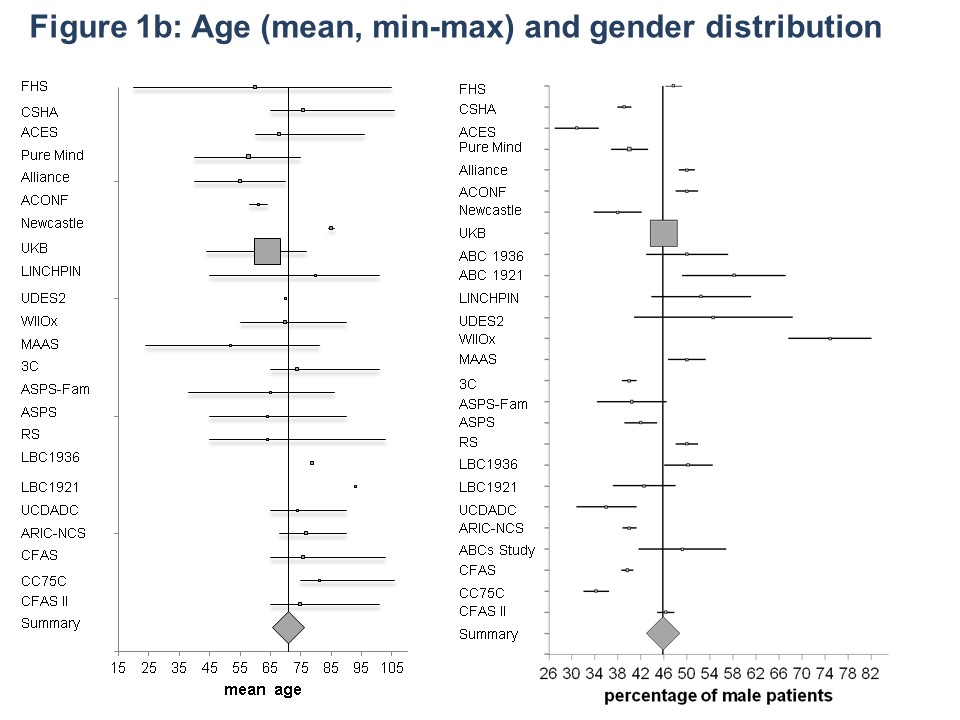

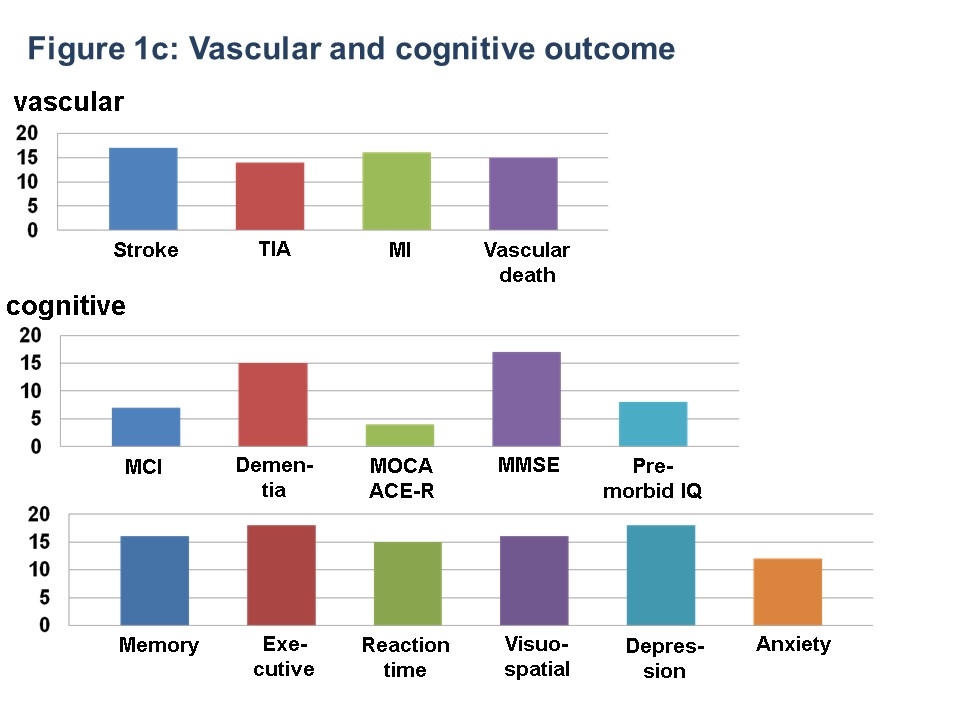

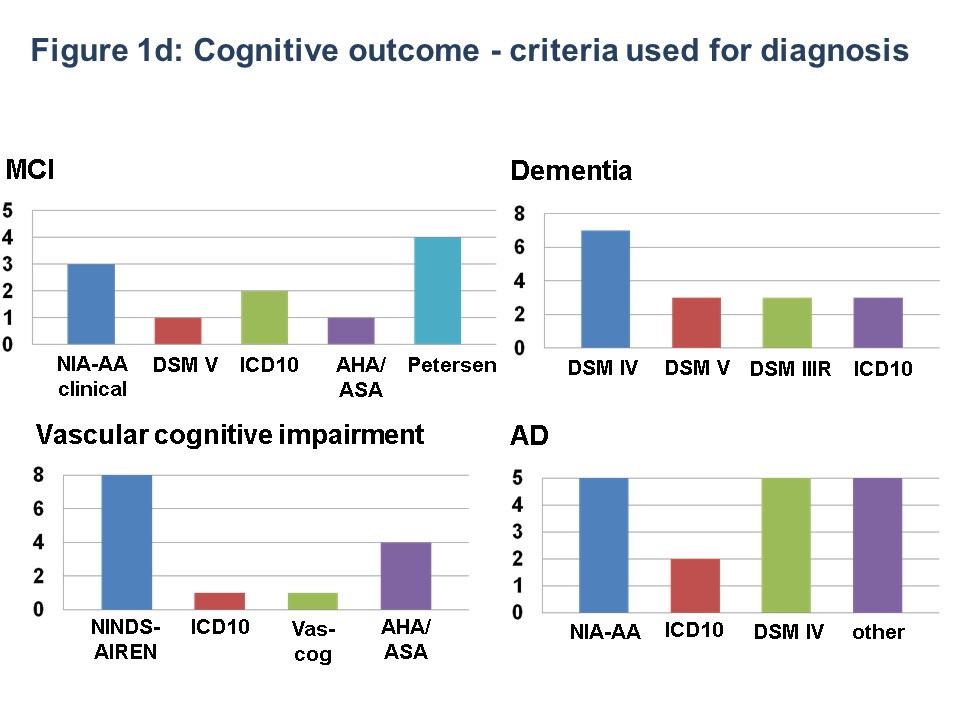


**Hospital-based cohorts recruited from stroke clinics (Supplementary Figure 2)**

Since stroke is the most important risk factor for vascular dementia (VaD), an important approach to examine the clinical profile and determinants of VaD has been the study of prospective cohorts of stroke patients. Beginning with the Stroke Data Bank Cohort in the early 1990s,[12] a number of studies have longitudinally examined patients with stroke or transient ischaemic attacks (TIAs) to identify the risk and protective factors for dementia, and the longitudinal course of cognitive and functional decline.[13] This approach has yielded many insights in this field. It helped establish that cognitive impairment and dementia are very common, and under-recognised, in stroke patients; the profile of VaD is different from Alzheimer’s Disease (AD), especially in its early stages, thereby challenging a uniform set of diagnostic criteria for both subtypes of dementia;[14] the majority of cognitive impairment due to cerebrovascular disease is below the threshold of dementia, but needs recognition if dementia-prevention is an objective, thereby leading to the introduction of the concept of VCI.[15] Small vessel disease (SVD) is a major determinant of post-stroke dementia.

Studies of post-stroke cognitive impairment have met with limitations. SO far, most studies have been small, with less than a few hundred participants.[13] The entry criteria have varied, with the majority restricting themselves to high-functioning ischaemic stroke patients who could participate in intensive investigations, and different studies have included TIAs, first-only strokes, or imposed other restrictions on the sample. The assessment tools have not been uniform, and the quality of neuroimaging has varied greatly. Not all studies performed baseline assessments to exclude individuals with pre-stroke cognitive impairment, and few have carefully tried to exclude concomitant AD pathology. Data on biomarkers of VaD, other than neuroimaging, have been scarce. Because of these limitations, many aspects of VaD and VCI remain poorly understood, and the additive and possible interactive effects of cerebrovascular and Alzheimer’s pathology largely unexplored. Some of the limitations in the literature can be overcome by bringing together various post-stroke studies of cognitive impairment in an international consortium.

Altogether the survey identified suitable hospital-based cohort studies: COGFAST, MSS 2, BIOSTROKE, STROKDEM, Sydney Stroke Study, CASPER, DEDEMAS/DEMDAS, STRIDE, and four studies without study name (see Supplementary Table 1: K. Abe; T. Quinn; B. Hee-Joon; A. Barugh). Eight of these studies have already completed recruitment and five of them have also completed follow-up. Nine studies included patients with any ischemic stroke/TIA as well as with haemorrhagic stroke. All patients were seen by stroke specialist for diagnosis and subtyping and the clinical diagnosis was supported by MRI in five studies, by CT in four studies and by a mixture of MRI and CT in 3 studies. TOAST was used by seven studies, OCSP by four studies and ASCO and other classifications by two studies, respectively. Two studies used TOAST and ASCO. Currently there are baseline data from 4134 patients available, including 3529 patients with imaging. The planned sample size for all studies together is 4702, including 4289 patients with imaging (Supplementary Figure 2a). Current mean age is 70 years and 56% of the patients were men (Supplementary Figure 2b). Baseline assessment covered relevant risk factors in all studies (e.g. hypertension, diabetes mellitus, hypercholestero­lemia, smoking, and medication) and education is missing in only one study. Brain imaging at baseline is missing in one study only. Eight studies performed MRI, one study CT, and 2 studies a mix of CT and MRI at baseline, in which all studies with MRI performed T2, eight studies T1, eight studies T2*, seven studies FLAIR and five studies DWI. DNA is stored in seven and blood in six studies. Nine studies had data at three months after the index event, in which one study (CASPER) had his baseline assessment 12 weeks after stroke. Nine studies had follow-up after 12 months and six studies after 36 and 60 months with one study (STRIDE) always shifted for three months. There is a large overlap in the methods used to measure functional, vascular and cognitive outcome (Supplementary Figure 2 c) and follow-up time points measuring these outcomes are well matched (see Figure 2, main paper). Furthermore, imaging at follow-up is performed by eight studies and two studies used PET imaging. Only one of these post stroke cohorts, COGFAST,[16] plus one other not included in the detailed analysis of incident post stroke dementia (LINCHPIN)[17]^,^[18] have collected post-mortem fixed and frozen brain tissue samples.

**Supplementary Figure 2 Hospital-based cohorts recruited from stroke clinics**


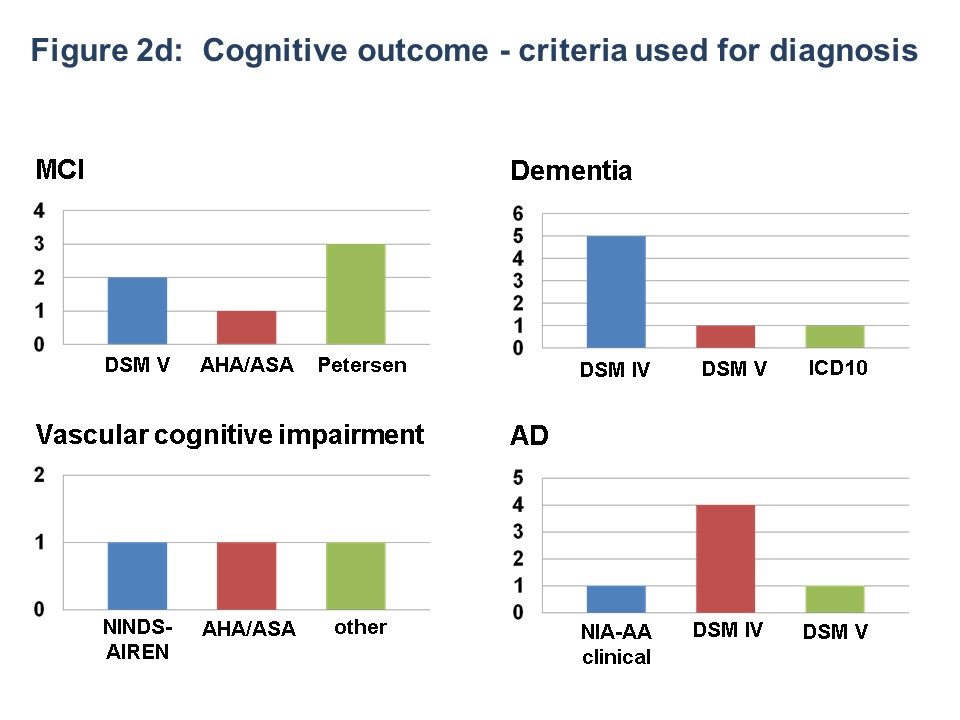

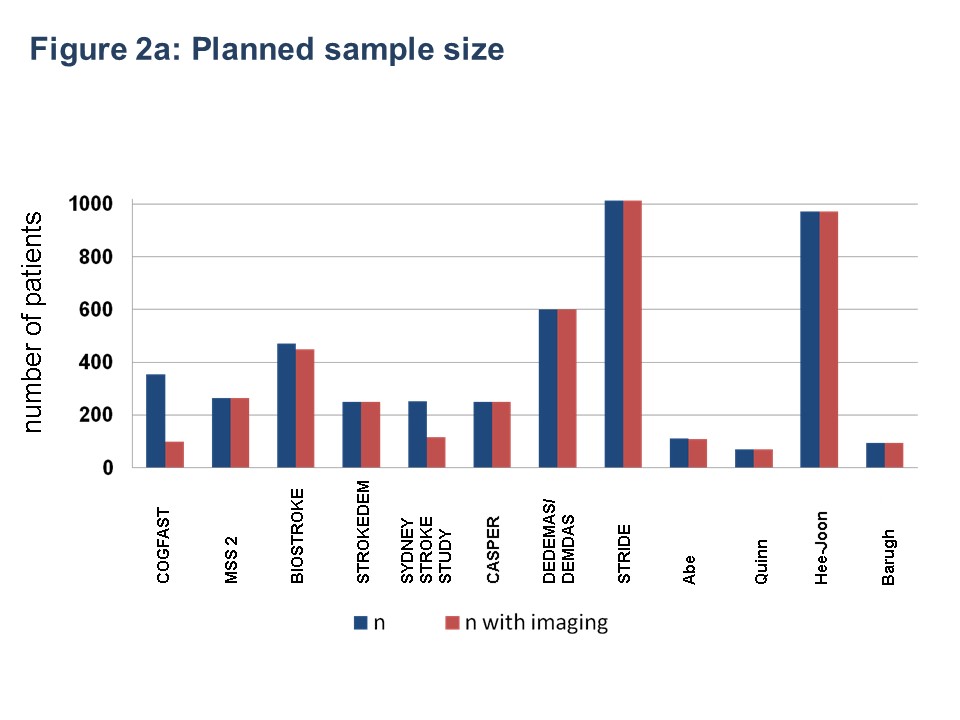

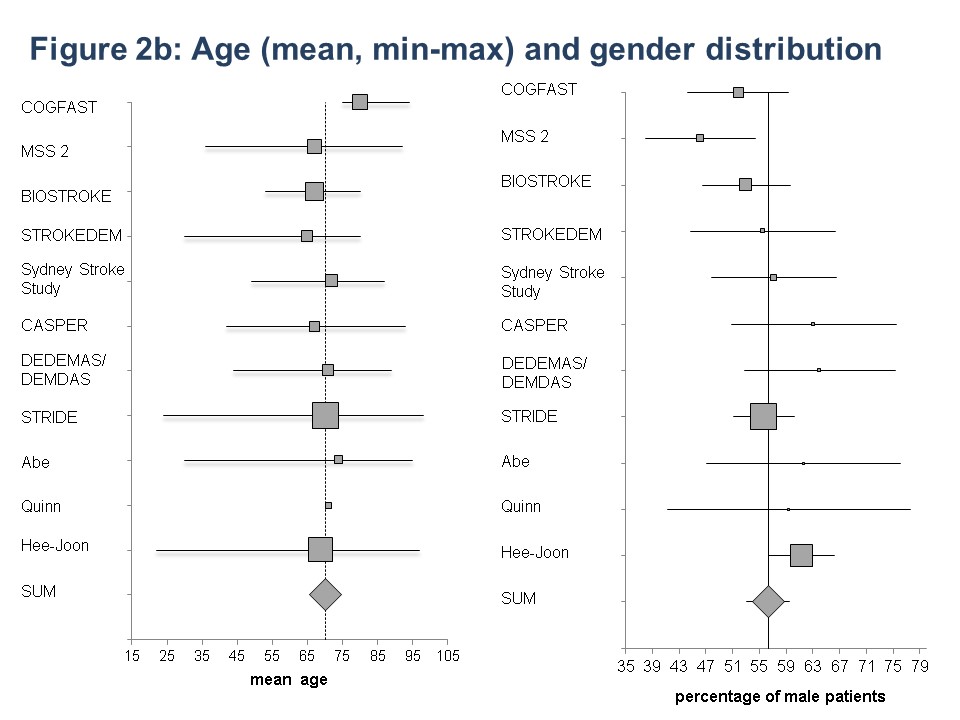

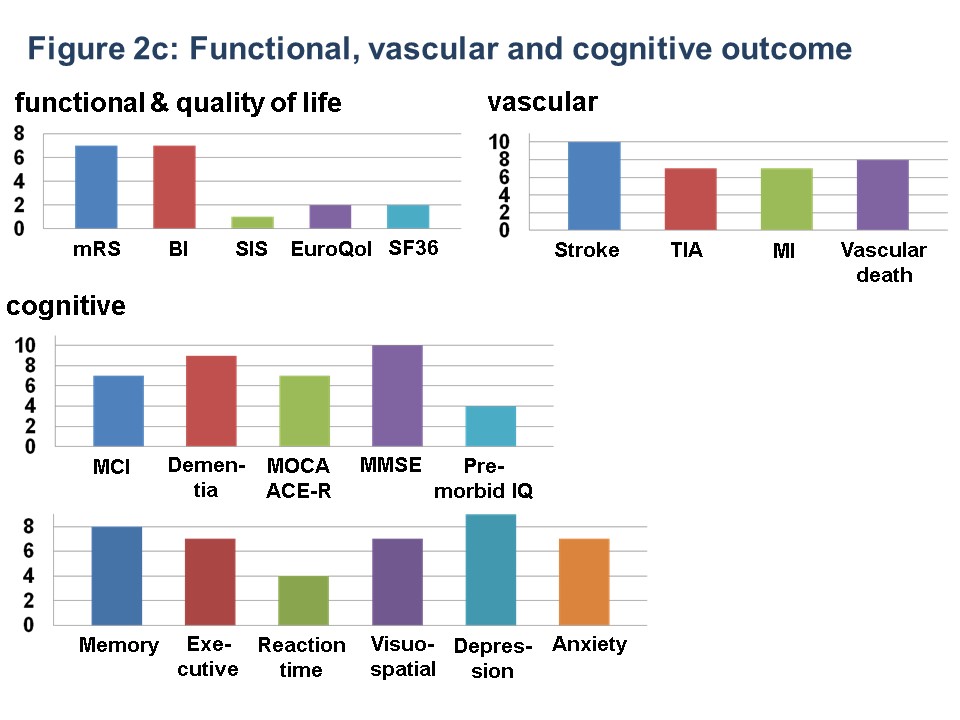


**Hospital-based cohorts recruited from memory clinics (Supplementary Figure 3)**

From a clinical perspective, patients attending a memory clinic represent a highly relevant population to study vascular contributions to cognitive decline and dementia. When in the diagnostic work-up of such patients reveals vascular lesions on brain imaging this generates questions such as: Are the lesions contributing to the cognitive profile in this patient? Are they relevant for prognosis? Should treatment be modified based on lesion presence or burden? Unfortunately, there is still limited evidence from diagnostic and prognostic studies and randomized controlled trials (RCTs) specifically in memory clinic based cohorts to answer these obvious questions. Extrapolation of observations from other cohorts, such as population based studies, may not be valid. Predictive factors for a disease in a relatively healthy population may not necessarily predict disease progression among individuals affected by the disease. Moreover, in the majority of patients attending a memory clinic vascular lesions co-occur with other pathologies, in particular Alzheimer-type processes. Different pathologies may have differential prognostic impact in different stages of the dementia process. For example, WMH burden is a stronger predictor of progressive neurodegeneration - i.e. progressive brain atrophy - in people with MCI and early Alzheimer’s disease than in later stages.[19,20] Co-occurring pathologies may also affect risk-benefit ratios of treatments that are typically used to reduce vascular risk, such as antithrombotic drugs. For example, RCTs testing aspirin in patients with Alzheimer’s disease observed rates of intracerebral haemorrhage that were much higher than those in people without Alzheimer’s disease.[21]

Altogether there are 15 hospital-based cohort studies based on memory clinic patients (AMPLE, NIMROD, VMCI-Tuscany, LADIS, MITNEC-C6, ONDRI, WMH in AD, String of Pearls, HBC, SuSpect-CAA, Harmy, CAA without ICH, VCI, TRACE-VCI (labled VCI-vidi in Figure), CREDOS, DELCODE; Supplementary Table 1). Furthermore, one study is recruiting AD-patients (WMH IN AD) and was therefore included in the following analysis. Six of these studies have more than one wave, eight have already completed the recruitment of the first or only wave and four of them have also completed follow-up. Currently there are baseline data from 19144 patients available, including 5982 patients with imaging. The planned sample size for all studies together is 21353, including 8153 patients with imaging (Supplementary Figure 3a). Current mean age is 73 years and 51% of the patients were men (Supplementary Figure 3b). Eight studies included patients with any cause of dementia or cognitive impairment, six studies patients with probable or definite vascular dementia or cognitive impairment, eight studies patients with probable or definite Alzheimer’s, and two studies patients with probable or definite Lewy body. Fourteen studies used dementia specialist for diagnosis and Supplementary Figure 3d shows the diagnosis criteria used. Baseline assessment covered relevant risk factors in all studies (e.g. hypertension, diabetes mellitus, hyper­cholestero­lemia, smoking, education). Information on medication is available from 15 studies, blood inflammatory markers from seven studies, and renal function from three studies. Eleven studies performed MRI and three studies a mix of CT and MRI at baseline, in which 14 studies performed T2, 12 studies T1, 10 studies T2*, 14 studies FLAIR and 7 studies DWI. DNA is stored in 13 and blood in 14 studies. Six studies had data after 1 month, four studies after 6 months, 14 studies after 12 months, one study after 18 months, nine studies after 24 months, seven studies after 36 months and one study after 48 months. There is a large overlap in the methods used to measure functional, vascular and cognitive outcome (Supplementary Figure 3d) and follow-up time points measuring these outcomes are well matched (see Figure 2 in main paper). Furthermore, imaging is repeatedly performed in 11 studies and three studies used PET imaging.

**Supplementary Figure 3 Hospital-based cohorts recruited from memory clinics (VCI-vidi is now called TRACE-VCI)**


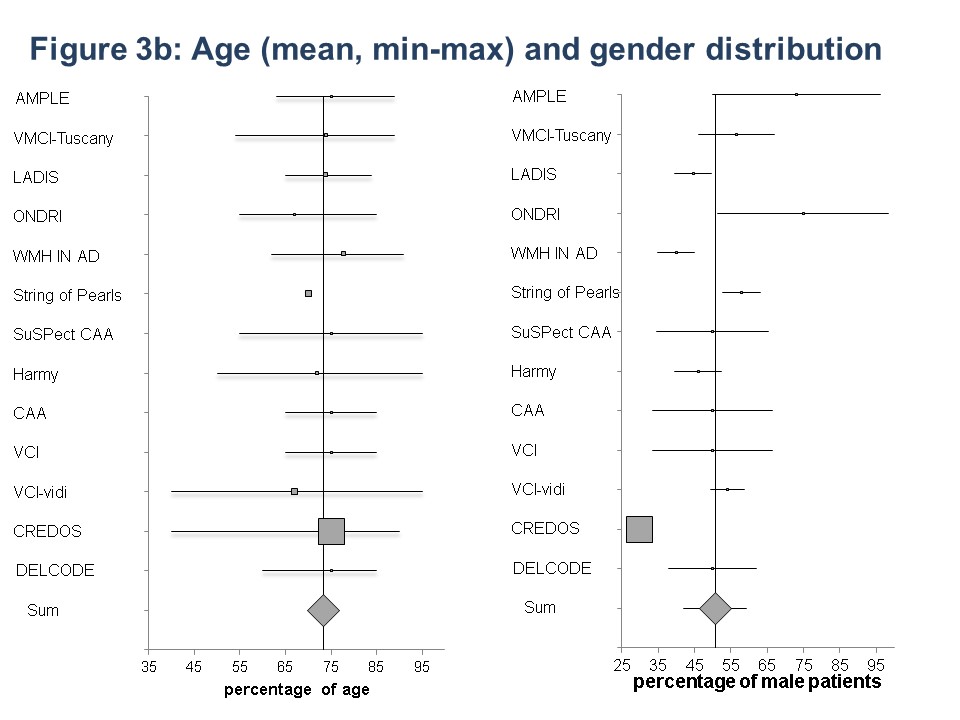

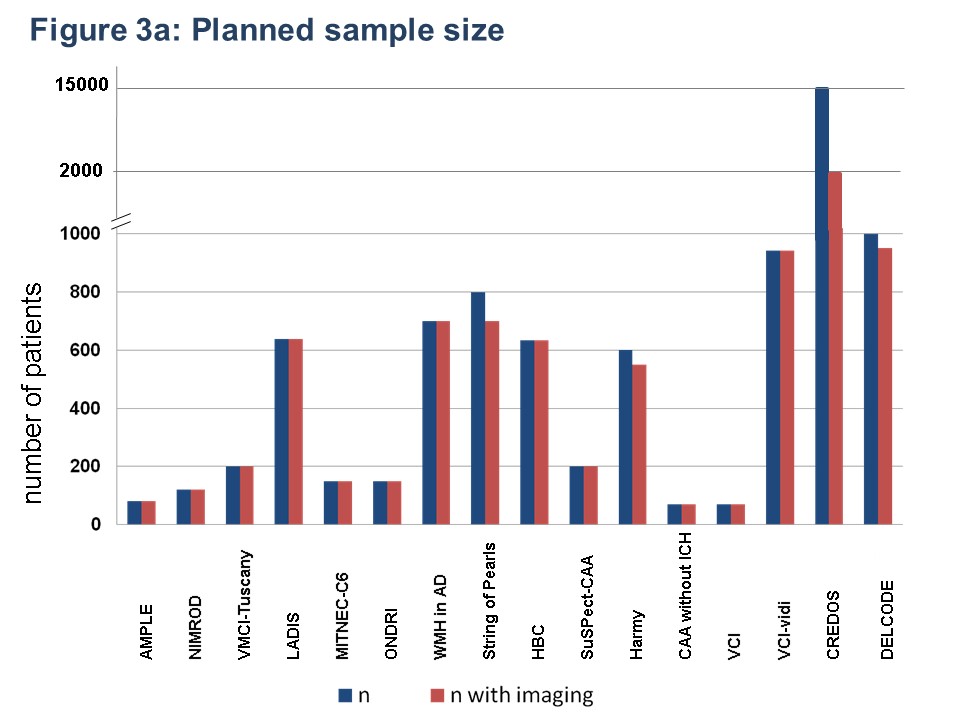

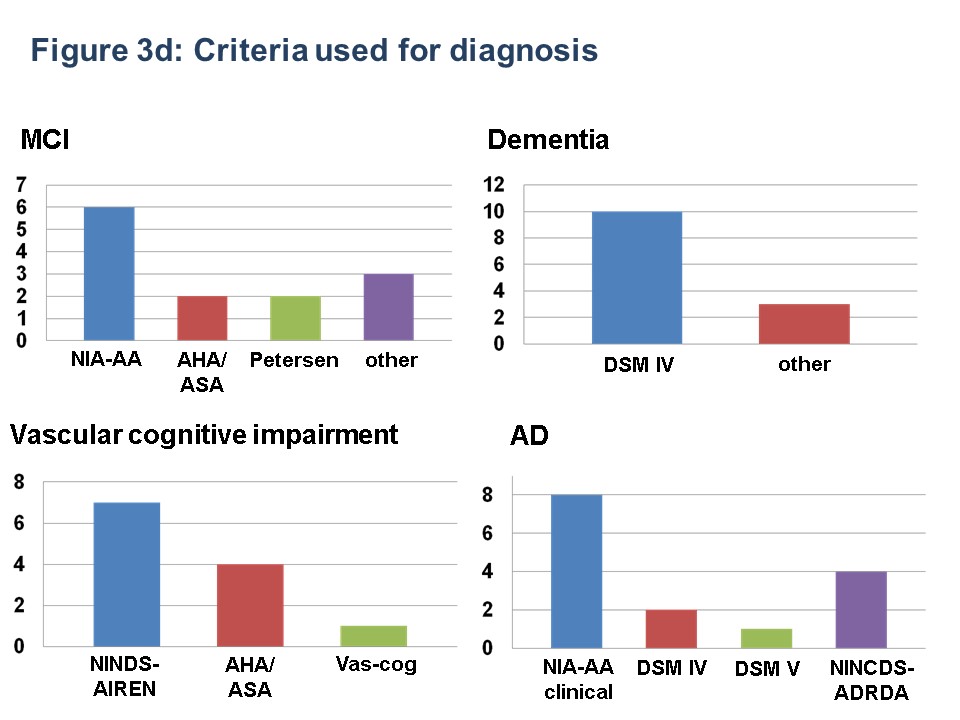

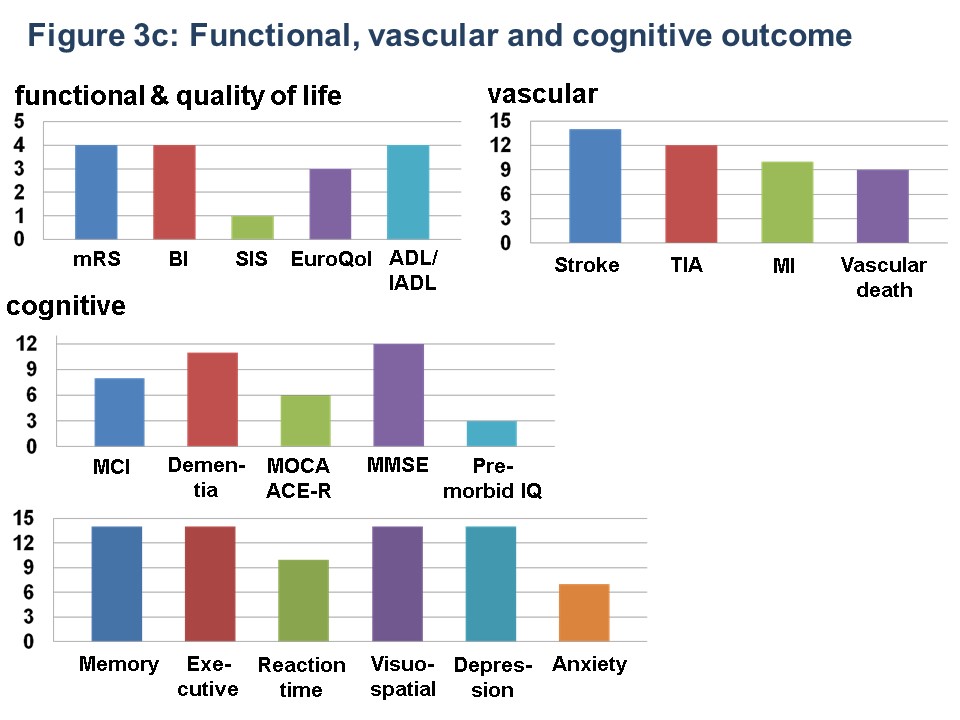


**Studies assessing gait and balance**

Gait and balance disorders are highly prevalent in the elderly population with rates that rapidly increase from around 15% at the age of 60 to 50% and more in ≥ 80 year old.^9-11^ They are associated with falls, greater risk of institutionalization and increased mortality and an often multifactorial aetiology.[22,23] Vascular cerebral disorders may also contribute to Parkinsonian symptoms.[24,25]

Gait and balance have rarely been an outcome variable in population based studies which examined the clinical consequences of cerebrovascular diseases thus far. A cross sectional analysis of participants in the leukoaraiosis and disability in the elderly (LADIS) study showed gait and balance performance to be correlated with the severity of white matter hyperintensities (WMH).[26] A 3-year follow up of these individuals documented overall deterioration of lower body motor function for the entire group but with large inter-individual variability both in the evolution of performance on gait and balance tests and their relation to WMH suggesting differences in compensatory capacity and the morphologic impact of vascular damage.[27] Analysis from the Radboud University Nijmegen diffusion tensor and MRI cohort (Run DMC Study) confirmed and extended these results by showing that WMH and lacunar infarcts where both independently associated with most gait parameters and stride length appeared to be the most sensitive parameter related to WMH severity.[28] Further investigations also showed an independent relation of gait disturbances with old cerebral microbleeds[29] and cortical thickness.^16,18^ Similarly in the Canadian Portion of the multinational Prospective Urban Rural Epidemiological (PURE) study, silent brain infarcts where associated with slower timed gait and lower volume of supratentorial white matter. Higher volume of supratentorial WMH was also associated with slower timed gait.^19^ Parallel associations where found with results on the digit symbol substitution test and support the interaction between cognitive and motor functioning. The Ibadan study on ageing which found that individuals with a more pronounced reduction in gait speed also experienced the worst cognitive decline.[30] WMH and their progression have been identified as independent predictors for subsequent falls.^21,22^ However, it is yet less clear what components of SVD beyond WMH are also contributory such as old subcortical infarcts microbleeds and brain atrophy. Also lesion topography may play a role.^23,24^

The survey found very limited information. Details were poorly captured in the questionnaire. There are six studies from general geriatric clinics but only one^22^ mentioned recording gait, walking or movement. Under "other", no study mentioned these words either. Seven studies mentioned somewhere in the questionnaire the word ‘gait’ (ABC1936, ABC1921, CASPER, ONDRI, PURE-MIND, RUNDMC, STRIDE). This suggests that problems of gait and balance are poorly assessed in vascular focused clinics for older people. A further study was identified after closure of the Survey, the Brain and Gait Study (London Canada).[3]^,^[4]^,^[5]^,^[6]^,^[7] Complexity certainly comes from the interaction of cerebral vascular causes with other neurologic disorders and non-neurologic causes which may give rise to dysfunction of gait and balance. In this context multimodal MRI (lesion identification and characterization, DTI, global and regional brain volumetry, etc.) might serve to disentangle the contributing roles of focal vs more diffuse (neurodegeneration) cerebral changes to the development of gait and balance disorders in future research (Table 4, main paper).

**Clinical Trials (Supplementary Figure 4)**

Altogether there are twelve clinical trials: three investigating the effect of blood pressure lowering after stroke (ENOS, RIGHT, RIGHT2), one the use of tranexamic acid in haemorrhagic stroke (TICH-2), one the use of fluoxetine (FOCUS), one the use of B vitamin (VITATOPS), two investigating the effect of exercise (Energl, PEXVP), all on functional recovery after stroke; one investigating the effect of pravastatin (PROSPER), one investigating secondary stroke prevention after lacunar stroke using antiplatelet therapy and intensive blood pressure control (SPS3 Trial), one investigating triple antiplatelet drugs in stroke prevention after TIA (TARDIS) and one testing intensive versus guideline statin, antiplatelet and blood pressure lowering to prevent cognitive decline after stroke (PODCAST).

Two of these trials have more than one wave, seven have already completed the recruitment of the first or only wave and five studies have also completed follow-up. One trial (Energl) will start in 2015. Currently there are baseline data from 20,035 patients available, including 12050 patients with imaging. The planned sample size for all trials together is 22,314 including 12,439 patients with imaging (Supplementary Figure 4a). Current mean age is 71.6 years and 58.5% of the patients were men (Supplementary Figure 4b). Ten trials recruited patients with ischemic stroke or TIA (PROSPER, SPS3, TICH-2, ENOS, FOCUS, VITATOPS, RIGHT, PODCAST, TARDIS, RIGHT2), one trial patients at risk of Alzheimer’s and healthy elderly (PEXVP) and one trail healthy elderly (Energl). Baseline assessment covered relevant risk factors in 10 trials (e.g. hypertension, diabetes mellitus, and hypercholesterolemia), smoking in nine trials, medication in eight trials and education in four trials. Blood inflammatory markers are available from two stroke trials, renal function from one stroke trial. Three trials performed MRI and five trials a mix of CT and MRI at baseline, all including T1, T2, T2* and FLAIR. DWI is available in three trials. DNA and blood is stored in the three trials. There is some overlap in the methods used to measure functional, vascular and cognitive outcome (Supplementary Figure 4). Follow-up time points and time of MRI imaging and outcome measurements is shown in Figure 2, main paper. Energl will use PET imaging at baseline.

**Supplementary Figure 4 Clinical Trials**


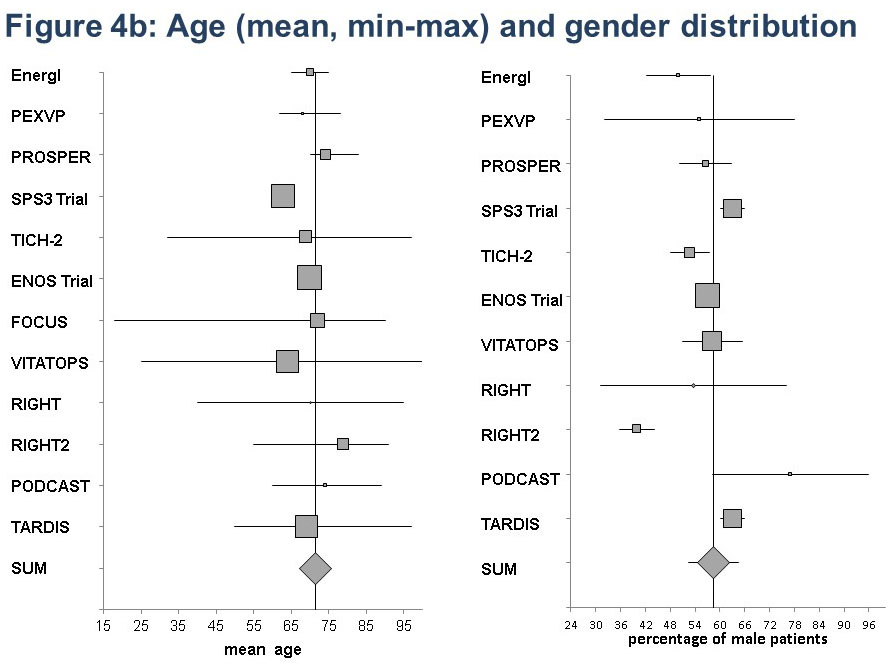

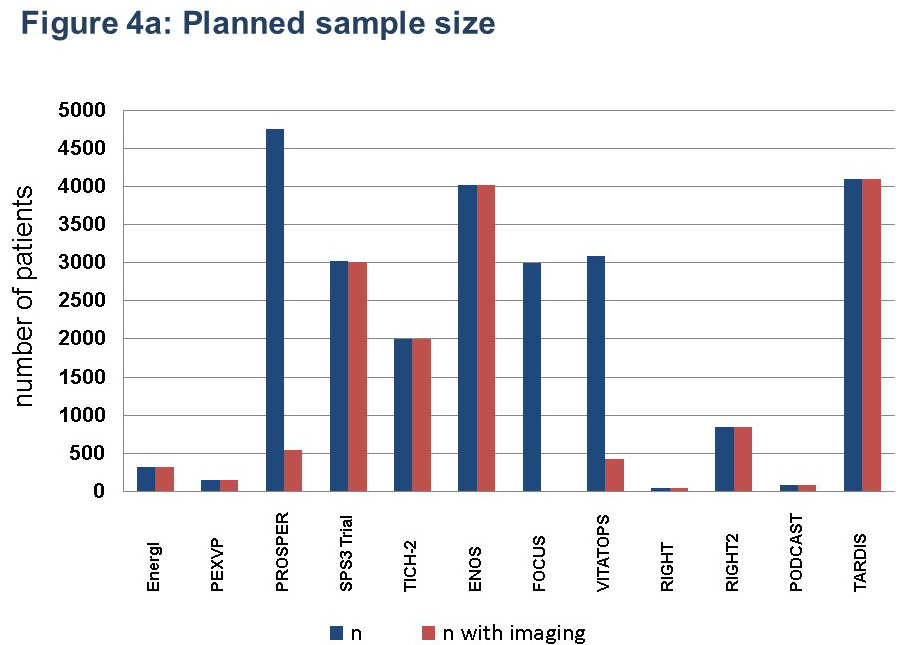

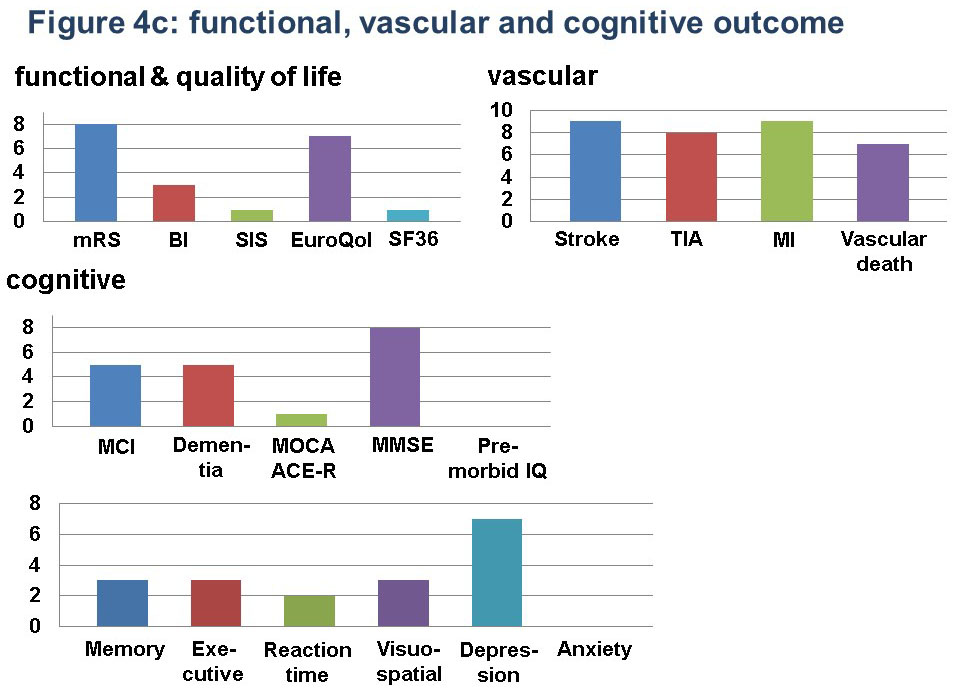


References

[1] Hankey, G. J., Ford, A. H., Yi, Q., Eikelboom, J. W., Lees, K. R., Chen, C. et al. Effect of B vitamins and lowering homocysteine on cognitive impairment in patients with previous stroke or transient ischemic attack: a prespecified secondary analysis of a randomized, placebo-controlled trial and meta-analysis. Stroke 2013;44:2232-2239.

[2] Cavalieri, M., Schmidt, R., Chen, C., Mok, V., de Freitas, G. R., Song, S. et al. B vitamins and magnetic resonance imaging-detected ischemic brain lesions in patients with recent transient ischemic attack or stroke: the VITAmins TO Prevent Stroke (VITATOPS) MRI-substudy. Stroke 2012;43:3266-3270.

[3] Montero-Odasso, M., Casas, A., Hansen, K. T., Bilski, P., Gutmanis, I., Wells, J. L. et al. Quantitative gait analysis under dual-task in older people with mild cognitive impairment: a reliability study. J Neuroeng Rehabil 2009;6:35.

[4] Muir, S. W., Speechley, M., Wells, J., Borrie, M., Gopaul, K., and Montero-Odasso, M. Gait assessment in mild cognitive impairment and Alzheimer's disease: the effect of dual-task challenges across the cognitive spectrum. Gait Posture 2012;35:96-100.

[5] Montero-Odasso, M., Muir, S. W., and Speechley, M. Dual-task complexity affects gait in people with mild cognitive impairment: the interplay between gait variability, dual tasking, and risk of falls. Arch Phys Med Rehabil 2012;93:293-299.

[6] Annweiler, C., Beauchet, O., Bartha, R., and Montero-Odasso, M. Slow gait in MCI is associated with ventricular enlargement: results from the Gait and Brain Study. J Neural Transm (Vienna) 2013;120:1083-1092.

[7] Montero-Odasso, M., Oteng-Amoako, A., Speechley, M., Gopaul, K., Beauchet, O., Annweiler, C. et al. The motor signature of mild cognitive impairment: results from the gait and brain study. J Gerontol A Biol Sci Med Sci 2014;69:1415-1421.

[8] Sposato, L. A., Coppola, M. L., Altamirano, J., Borrego Guerrero, B., Casanova, J., De Martino, M. et al. Program for the epidemiological evaluation of stroke in Tandil, Argentina (PREVISTA) study: rationale and design. Int J Stroke 2013;8:591-597.

[9] Akinyemi, R. O., Allan, L., Owolabi, M. O., Akinyemi, J. O., Ogbole, G., Ajani, A. et al. Profile and determinants of vascular cognitive impairment in African stroke survivors: the CogFAST Nigeria Study. J Neurol Sci 2014;346:241-249.

[10] Akinyemi, R. O., Firbank, M., Ogbole, G. I., Allan, L. M., Owolabi, M. O., Akinyemi, J. O. et al. Medial temporal lobe atrophy, white matter hyperintensities and cognitive impairment among Nigerian African stroke survivors. BMC Res Notes 2015;8:625.

[11] Gorelick, P. B., Scuteri, A., Black, S. E., DeCarli, C., Greenberg, S. M., Iadecola, C. et al. Vascular contributions to cognitive impairment and dementia: a statement for healthcare professionals from the American Heart Association/American Stroke Association. Stroke 2011;42:2672-2713.

[12] Tatemichi, T. K., Foulkes, M. A., Mohr, J. P., Hewitt, J. R., Hier, D. B., Price, T. R. et al. Dementia in stroke survivors in the Stroke Data Bank cohort. Prevalence, incidence, risk factors, and computed tomographic findings. Stroke 1990;21:858-866.

[13] Gottesman, R. F. and Hillis, A. E. Predictors and assessment of cognitive dysfunction resulting from ischaemic stroke. Lancet Neurol 2010;9:895-905.

[14] Sachdev, P., Kalaria, R., O'Brien, J., Skoog, I., Alladi, S., Black, S. E. et al. Diagnostic criteria for vascular cognitive disorders: a VASCOG statement. Alzheimer Dis Assoc Disord 2014;28:206-218.

[15] Hachinski, V. Vascular dementia: a radical redefinition. Dementia 1994;5:130-132.

[16] Allan, L. M., Rowan, E. N., Firbank, M. J., Thomas, A. J., Parry, S. W., Polvikoski, T. M. et al. Long term incidence of dementia, predictors of mortality and pathological diagnosis in older stroke survivors. Brain 2011;134:3713-3724.

[17] Samarasekera, N., Al-Shahi Salman, R., Huitinga, I., Klioueva, N., McLean, C. A., Kretzschmar, H. et al. Brain banking for neurological disorders. Lancet Neurol 2013;12:1096-1105.

[18] Samarasekera, N., Lerpiniere, C., Fonville, A., Farrall, A., Wardlaw, J., White, P. et al. Consent for brain tissue donation after intracerebral haemorrhage: a community-based study. PLoS ONE 2015;10:e0135043.

[19] Barnes, J., Carmichael, O. T., Leung, K. K., Schwarz, C., Ridgway, G. R., Bartlett, J. W. et al. Vascular and Alzheimer's disease markers independently predict brain atrophy rate in Alzheimer's Disease Neuroimaging Initiative controls. Neurobiol Aging 2013;34:1996-2002.

[20] Kandiah, N., Chander, R. J., Ng, A., Wen, M. C., Cenina, A. R., and Assam, P. N. Association between white matter hyperintensity and medial temporal atrophy at various stages of Alzheimer's disease. Eur J Neurol 2015;22:150-155.

[21] Thoonsen, H., Richard, E., Bentham, P., Gray, R., van Geloven, N., De Haan, R. J. et al. Aspirin in Alzheimer's disease: increased risk of intracerebral hemorrhage: cause for concern? Stroke 2010;41:2690-2692.

[22] Snijders, A. H., van de Warrenburg, B. P., Giladi, N., and Bloem, B. R. Neurological gait disorders in elderly people: clinical approach and classification. Lancet Neurol 2007;6:63-74.

[23] Verghese, J., LeValley, A., Hall, C. B., Katz, M. J., Ambrose, A. F., and Lipton, R. B. Epidemiology of gait disorders in community-residing older adults. J Am Geriatr Soc 2006;54:255-261.

[24] Buchman, A. S., Leurgans, S. E., Nag, S., Bennett, D. A., and Schneider, J. A. Cerebrovascular disease pathology and parkinsonian signs in old age. Stroke 2011;42:3183-3189.

[25] de Laat, K. F., van Norden, A. G., Gons, R. A., van Uden, I. W., Zwiers, M. P., Bloem, B. R. et al. Cerebral white matter lesions and lacunar infarcts contribute to the presence of mild parkinsonian signs. Stroke 2012;43:2574-2579.

[26] Baezner, H., Blahak, C., Poggesi, A., Pantoni, L., Inzitari, D., Chabriat, H. et al. Association of gait and balance disorders with age-related white matter changes: the LADIS study. Neurology 2008;70:935-942.

[27] Kreisel, S. H., Blahak, C., Bazner, H., Inzitari, D., Pantoni, L., Poggesi, A. et al. Deterioration of gait and balance over time: the effects of age-related white matter change - the LADIS study. Cerebrovasc Dis 2013;35:544-553.

[28] de Laat, K. F., van Norden, A. G., Gons, R. A., van Oudheusden, L. J., van Uden, I. W., Bloem, B. R. et al. Gait in elderly with cerebral small vessel disease. Stroke 2010;41:1652-1658.

[29] de Laat, K. F., van den Berg, H. A., van Norden, A. G., Gons, R. A., Olde Rikkert, M. G., and de Leeuw, F. E. Microbleeds are independently related to gait disturbances in elderly individuals with cerebral small vessel disease. Stroke 2011;42:494-497.

[30] Ojagbemi, A., D'Este, C., Verdes, E., Chatterji, S., and Gureje, O. Gait speed and cognitive decline over 2 years in the Ibadan study of aging. Gait Posture 2015;41:736-740.
