# Supplementary material for: METACOHORTS for the study of vascular disease and its contribution to cognitive decline and neurodegeneration: An initiative of the Joint Programme for Neurodegenerative Disease Research
Source: Alzheimers Dement. 2016 Dec;12(12):1235–49. doi: 10.1016/j.jalz.2016.06.004 (PMC5399602; doi:10.1016/j.jalz.2016.06.004)
Supplement: Appendix [file mmc2.pdf]

Realising the potential of cohort studies to determine the vascular

Introduction

This survey will ask you about:

- Principle study characteristics
- Study population characteristics
- Clinical assessments at recruitment
- Biomarkers
- Imaging and related baseline assessments (follow-up imaging recorded on outcomes form)
- Outcomes
- Perceived strengths and weakness of cohort (suitability for analyses)

Approximate values (e.g. for average age or number/proportion of males) are fine.

You can move through the questions using the Tab key.

To enter additional studies, please email k.shuler@ed.ac.uk and request a new link.

Details of person completing data entry

Surname

First Name

Institution

City

Country

Email address

Telephone number

Principal study characteristics

Realising the potential of cohort studies to determine the vascular

Name of cohort

Abbreviation/Acronym

Principal Investigator

Surname

First Name

Original purpose of study (Primary aim)

Recruitment and completion timelines

Is there more than one phase or wave of recruitment?

- Yes
- No

1st or only phase/wave of recruitment

Year recruitment began (YYYY)

Recruitment of 1st or only phase completed?

- Yes
- No

If yes, what year was it completed? (YYYY)

If no, what year is it expected by? (YYYY)

Follow-up of 1st or only phase completed?

- ◯ Yes
- ◯ No
- ◯ Not applicable (no follow-up)

If yes, what year was it completed? (YYYY)

If no, what year is it expected by? (YYYY)

2nd phase or wave (please skip if not applicable)

Year 2nd phase recruitment began (YYYY)

If more than 1 phase, has 2nd phase completed?

- ◯ Yes
- ◯ No

If yes, what year was it completed? (YYYY)

If no, what year is it expected by? (YYYY)

Follow-up of 2nd phase completed?

- ◯ Yes
- ◯ No
- ◯ Not applicable (no follow-up)

If yes, what year was it completed? (YYYY)

If no, what year is it expected by? (YYYY)

Is there a 3rd phase or wave?

- ◯ Yes
- ◯ No

Principal study characteristics continued

Realising the potential of cohort studies to determine the vascular

Type of research

- ◯ Clinical trial
- ◯ Observational study

If observational

If observational

- ◯ Cross-sectional
- ◯ Longitudinal

If longitudinal observational or a clinical trial

If longitudinal observational or a clinical trial, please indicate the assessment timepoints (months) whether face to face or postal/telephone, scanning or other assessments (tick all that apply)

- ☐ 1
- ☐ 3
- ☐ 6
- ☐ 12
- ☐ 18
- ☐ 24
- ☐ 36
- ☐ 48
- ☐ 60
- ☐ Other (please specify)

Principal study characteristics continued

Setting

- ◯ Hospital/Clinic Based
- ◯ Community via advertising or other method
- ◯ Community with population-based sampling
- ◯ Hospital/Clinic-based for some recruits (e.g. stroke patients) and Community-based for others (e.g. controls)

Realising the potential of cohort studies to determine the vascular

Was/is study

- ○ Single centre
- ○ Multi-centre

Recruitment from

- ☐ Any healthy volunteers
- ☐ Subjects randomly selected from a geographical population
- ☐ Stroke or TIA clinic
- ☐ Memory clinic
- ☐ General geriatric clinic
- ☐ Other (please specify)

Interventions (if relevant)

Clinical trials ID# (if applicable)

Funders

Please provide reference to one (1) key publication related to cohort

1st author

Title

Journal

Volume

Pages

Year

PubMed ID

Study website (if available)

Please indicate which of the following data types have been collected (note details for these domains are collected on later pages where relevant)

- ☐ Baseline clinical assessment
- ☐ Any neuroimaging (MRI or CT)
- ☐ Cognition (baseline or follow-up)
- ☐ Long-term outcomes (any type)
- ☐ Biological samples including DNA or other genetic samples
- ☐ Other imaging (retinal) or physiological measures

Are you/your collaborators interested in using your cohort's data for

- ☐ Data sharing
- ☐ Recall for new data collection
- ☐ Sharing blood and/or genetic samples available for analysis

Apart from administrative approvals, do you foresee any major practical problems or specific restrictions for using your data for (if yes, please explain in comment below)

- ☐ Sharing of numeric data
- ☐ Sharing of images
- ☐ Sharing of biological samples
- ☐ Further follow up being performed on your cohort
- ☐ No major problems forseen

Please explain your response if relevant

Study population characteristics

Total number in cohort (A)

At present

With neuroimaging

If recruitment ongoing, total number expected (B)

In cohort

With neuroimaging

If not applicable, please enter "N/A"

Age (if ongoing, put current minimum/maximum)

Minimum (approximate)

Maximum (approximate)

Average (approximate)

SD

Males

Number (approximate)

Percentage

Are you recruiting people with

- [ ] Stroke or TIA
- [ ] Cognitive impairment or dementia
- [ ] Healthy ageing
- [ ] Specific genetic group
- [ ] Other (please specify)

If stroke

If stroke, please tick all that apply

- [ ] Not applicable
- [ ] Any ischaemic stroke or TIA
- [ ] Haemorrhagic stroke
- [ ] Any anterior circulation ischaemic stroke only
- [ ] Any posterior circulation ischaemic stroke only
- [ ] Lacunar stroke only
- [ ] Large artery stroke only
- [ ] Cardioembolic only
- [ ] Other (please specify)

If cognitive impairment or dementia

If cognitive impairment or dementia, please tick all that apply

- [ ] Not applicable
- [ ] Any cause of dementia or cognitive impairment
- [ ] Probable or definite vascular
- [ ] Probable or definite Alzheimer's
- [ ] Probable or definite Lewy body
- [ ] Other (please specify)

Clinical assessments at recruitment - stroke

Not applicable

- Yes
- No

All patients seen by stroke specialist for diagnosis +/-subtyping?

- Yes
- No

The diagnosis is based only on case records/other non-specialist diagnosis?

- Yes
- No

The clinical diagnosis is supported by brain imaging?

- Yes
- No

If yes, please indicate if

- MRI in all or most
- CT in all or most
- Either (ie a mixture)

A stroke classification is used?

- ◯ Yes
- ◯ No

If yes, tick all that apply

- ☐ TOAST
- ☐ OCSP
- ☐ CCS
- ☐ ASCO
- ☐ None
- ☐ Not relevant
- ☐ Other (please specify)

Clinical assessments at recruitment - cognitive impairment/dementia

Not applicable

- ◯ Yes
- ◯ No

All patients are diagnosed by specialist in dementia?

- ◯ Yes
- ◯ No

Diagnosis is based only on case records or other non-specialist diagnosis?

- ◯ Yes
- ◯ No

An MCI or dementia classification is used

- ◯ Yes
- ◯ No

If yes, please tick all that apply

Criteria used for MCI

- [ ] NIA-AA
- [ ] DSM V
- [ ] ICD10
- [ ] AHA/ASA (2011)
- [ ] None
- [ ] Not relevant
- [ ] Other (e.g. modified Petersen criteria)

Criteria used for dementia

- [ ] DSM IV
- [ ] DSM V
- [ ] ICD10
- [ ] None
- [ ] Not relevant
- [ ] Other (please specify)

Criteria used to assign vascular cognitive impairment

- [ ] NINDS-AIREN
- [ ] ICD10
- [ ] Vas-cog
- [ ] AHA/ASA (2011)
- [ ] None
- [ ] Not relevant
- [ ] Other (please specify)

Criteria used to assign AD

- [ ] NIA-AA Clinical
- [ ] NIA-AA with supporting biomarkers
- [ ] ICD10
- [ ] DSM IV
- [ ] DSM V
- [ ] IWG-2
- [ ] IWG-2 with supporting pathophysiological biomarker support
- [ ] None
- [ ] Not relevant

Other (please specify)

If AD was assessed were amyloid-specific biomarkers used for diagnosis?

- ( ) Yes
- ( ) No
- ( ) Not relevant

If yes, was this with

- [ ] PET imaging
- [ ] CSF

What cognition assessment method(s) was used

- [ ] MOCA
- [ ] ACE-R or equivalent
- [ ] MMSE
- [ ] IQCODE for (e.g. pre-stroke) cognitive impairment
- [ ] NART or equivalent test of premorbid (crystallised) intelligence
- [ ] Detailed memory tests
- [ ] Detailed executive function tests
- [ ] Reaction time tests
- [ ] Visuospatial tests
- [ ] Other (please specify)

Clinical assessments continued

Mental health assessed? (e.g. depression or anxiety)

- ◯ Yes
- ◯ No

Vascular risk factors collected?

- ◯ Yes
- ◯ No

If yes, please tick all that apply

- ☐ Hypertension
- ☐ Diabetes Mellitus
- ☐ Hypercholesterolemia
- ☐ Smoking
- ☐ Salt or other dietary factors
- ☐ Other (please specify)

Medications recorded?

- ◯ Yes
- ◯ No

If yes, please indicate if

- ☐ At baseline
- ☐ At follow-up (if applicable)

Educational attainment recorded?

- ◯ Yes
- ◯ No

Socioeconomic status recorded?

- ◯ Yes
- ◯ No

If yes

	Yes	No
Current	◯	◯
Childhood	◯	◯

Biomarkers

DNA stored?

◯ Yes

◯ No

Biomarkers collected? (i.e. blood, haematology, biochemistry, urine, renal function, CSF)

◯ Yes

◯ No

Biomarkers continued

Please respond to these questions if any of the following were assessed, even if only on a subset of patients.

Blood samples stored?

◯ Yes

◯ No

If yes

☐ Serum

☐ Plasma

☐ Stored at -80

☐ Stored at -20

Haematology

- [ ] Hb
- [ ] Hct
- [ ] Full Blood Count
- [ ] White Cell Count
- [ ] B12
- [ ] Folate
- [ ] Other (please specify)

Biochemistry

- [ ] Na
- [ ] K
- [ ] Cl
- [ ] Urea
- [ ] Liver function tests
- [ ] Cholesterol
- [ ] Triglycerides
- [ ] Homocysteine
- [ ] Other (please specify)

CSF - did you collect and analyse CSF?

- ○ Yes
- ○ No

CSF - do you have stored samples?

- ○ Yes
- ○ No

Renal function

- ○ Yes
- ○ No

Urine specimens analysed?

- ◯ Yes
- ◯ No

Urine stored for further analyses (e.g. proteomics)?

- ◯ Yes
- ◯ No

Blood inflammatory markers analysed?

- ◯ Yes
- ◯ No

Imaging and related baseline assessment measurements (follow-up imaging rec...

Brain imaging or other technical assessments (e.g. endothelial function, detailed BP, etc.) collected at baseline?
(Note: such assessments performed during follow-up can be recorded on the "Outcomes" section)

- ◯ Yes
- ◯ No

Imaging and related baseline assessment measurements continued

Type of brain imaging at baseline (tick closest match)

- ◯ CT
- ◯ MRI
- ◯ Mix of CT and MRI

If MR Brain imaging (tick all that apply)

Diagnostic structural MRI

- [ ] T1
- [ ] FLAIR
- [ ] T2
- [ ] T2*
- [ ] DWI
- [ ] Angiography

3D sequence

- [ ] T1
- [ ] FLAIR

Advanced MRI

- [ ] DTI
- [ ] fMRI
- [ ] ASL
- [ ] Permeability
- [ ] SWI-mineral
- [ ] Magnetisation Transfer Imaging
- [ ] Advanced structural
- [ ] Cerebrovascular reactivity
- [ ] Other (please specify)

MRI quality control

- [ ] Visual review
- [ ] Phantom
- [ ] 'Human' phantom
- [ ] None
- [ ] Not applicable (i.e. no MRI)
- [ ] Other (please specify)

Imaging of other organs (retinal/carotid/cardiac) affected by vascular disease?

- ◯ Yes
- ◯ No

If yes, state modality

- ☐ Retinal imaging
- ☐ US
- ☐ MRI
- ☐ CT
- ☐ Not relevant
- ☐ Other (please specify)

Physiological measures

- ☐ Detailed BP
- ☐ Ankle Brachial Pressure Index
- ☐ Pulse Wave Velocity
- ☐ Forced Expiratory Volume
- ☐ ECG
- ☐ Other (please specify)

Dynamic endothelial function

- ☐ Forearm blood flow
- ☐ TCD with challenge
- ☐ Cerebrovascular reactivity with BOLD
- ☐ Other (please specify)

Analysed data available

Brain volumes

- ◯ Yes
- ◯ No

If yes, please tick all that apply

- [ ] ICV
- [ ] CSF volume
- [ ] Whole brain volume
- [ ] Grey matter volume
- [ ] White matter volume
- [ ] WMH volume
- [ ] Infarct volume
- [ ] Hippocampal volume
- [ ] Ventricular volume
- [ ] Lobar volumes
- [ ] DTI parameters/tracts
- [ ] Other (please specify)

Visual score of brain appearance

- ( ) Yes
- ( ) No

If yes, please tick all that apply

- [ ] Atrophy global
- [ ] Atrophy regional
- [ ] WMH
- [ ] Lacunes
- [ ] Cortical infarcts
- [ ] Microbleeds
- [ ] Haemorrhages

Outcomes

Outcomes assessed

- ( ) Yes
- ( ) No

Outcomes continued

Functional outcome or quality of life assessed?

- [ ] modified Rankin Scale
- [ ] Oxford Handicap Scale
- [ ] Barthel Index
- [ ] Stroke Impact Scale
- [ ] Other functional (please specify in "Other comment box")
- [ ] EuroQol (e.g. EQ-5D)
- [ ] Other quality of life (please specify in "Other comment box")

Other (please specify)

Functional outcome or quality of life - Interval(s) collected (months)

- [ ] 1
- [ ] 3
- [ ] 6
- [ ] 12
- [ ] 24
- [ ] 36
- [ ] 48
- [ ] 60
- [ ] >60
- [ ] Other (please specify)

Vascular

- [ ] None/Not applicable
- [ ] Stroke
- [ ] TIA
- [ ] MI
- [ ] Vascular death
- [ ] Other (please specify)

Vascular - Interval(s) collected (months)

- [ ] 1
- [ ] 3
- [ ] 6
- [ ] 12
- [ ] 24
- [ ] 36
- [ ] 48
- [ ] 60
- [ ] >60
- [ ] Other (please specify)

Cognitive

- [ ] None/Not applicable
- [ ] Incident mild cognitive impairment
- [ ] Incident dementia
- [ ] MOCA/ACE-R or equivalent
- [ ] MMSE
- [ ] Pre-morbid IQ
- [ ] Detailed memory
- [ ] Detailed executive
- [ ] Reaction time
- [ ] Visuospatial
- [ ] Depression
- [ ] Anxiety
- [ ] Other (please specify)

Cognitive - Interval(s) collected (months)

- [ ] 1
- [ ] 3
- [ ] 6
- [ ] 12
- [ ] 24
- [ ] 36
- [ ] 48
- [ ] 60
- [ ] >60
- [ ] Other (please specify)

Neuroimaging follow-up

- [ ] None/not applicable
- [ ] CT
- [ ] Structural MRI (eg axial/sagittal T1, T2, FLAIR, T2*)
- [ ] 3D MRI sequences (eg T1, FLAIR, T2)
- [ ] DTI
- [ ] fMRI
- [ ] Perfusion
- [ ] Other (please specify)

Neuroimaging follow-up - Interval(s) collected (months)

- [ ] 1
- [ ] 3
- [ ] 6
- [ ] 12
- [ ] 24
- [ ] 36
- [ ] 48
- [ ] 60
- [ ] >60
- [ ] Other (please specify)

Is your cohort population thought to be representative of patients or volunteers in the source population from whom your cohort is recruited?

- ○ Yes
- ○ No

Do you collect data on non-recruited patients or subjects so as to provide evidence of the representativeness of your cohort to the source population and generalisability of your cohort to other relevant populations? (This might for example include a registry of all cases or centralised health care records)?

- ◯ Yes
- ◯ No

What are the main reasons for losses to follow-up (tick all that apply)?

- ☐ Death
- ☐ Recurrent stroke
- ☐ Dementia
- ☐ Unable to attend for examination due to physical impairment
- ☐ Unwilling to continue follow-up
- ☐ Moved away
- ☐ Other (please specify)

Final Questions

Perceived strengths of cohort (e.g. what types of analysis do you consider the data suitable for?)

Perceived weaknesses of cohort (types of analyses that would not be appropriate/that the data would not support)

Thank you very much for your time

The Joint Programme Of Neurodegeneration Working Group On Vascular Contributions To Neurodegeneration

CIHR/DZNE/MRC
